# Genome-wide association studies and candidate gene identification under salinity stress in bread wheat (*Triticum aestivum* L.)

**DOI:** 10.3389/fpls.2026.1817999

**Published:** 2026-04-22

**Authors:** B. Jagadhesan, Harish Chandra Singh, Sujata Thakur, Juhee Kumari, Sundeep Kumar, Shailendra Kumar Jha, Jyoti Kumari, Arvind Kumar, Rakesh Singh, Lekshmy Sathee, Gyanendra Pratap Singh, Amit Kumar Singh

**Affiliations:** 1 Division of Plant Physiology, Indian Council of Agricultural Research (ICAR)-Indian Agricultural Research Institute, New Delhi, India; 2 Indian Council of Agricultural Research (ICAR)-National Bureau of Plant Genetic Resources, New Delhi, India; 3 Department of Genetics and Plant Breeding, Sam Higginbottom University of Agriculture, Technology and Sciences, Prayagraj, Uttar Pradesh, India; 4 Division of Genetics, Indian Council of Agricultural Research (ICAR)-Indian Agricultural Research Institute, New Delhi, India; 5 Indian Council of Agricultural Research (ICAR)-Central Soil Salinity Research Institute, Karnal, Haryana, India

**Keywords:** bread wheat, candidate genes, GWAS, salinity, salt-tolerance

## Abstract

Salinity is a major abiotic stress in wheat production across the globe, especially in arid and semi-arid areas. In this study, 313 genetically diverse wheat genotypes were assessed for vegetative-stage salinity tolerance in a hydroponic condition and genotyped using 35K Axiom SNP array. Genotyping of the 313 wheat genotypes produced 24,968 polymorphic SNPs. To dissect salt tolerance, marker-trait association analysis was carried out using the salt tolerance indices of three traits, such as leaf chlorophyll content, green leaf area, and dry biomass. In total, 24 quantitative trait nucleotides (QTNs) showing significant associations with these three traits were identified, including seven linked to leaf chlorophyll content, twelve with green leaf area, and five to dry biomass traits. Four QTNs (Q.CCI-E3-1A, Q.CCI-E3-5D, Q.DB-E3-1B, and Q.GLA-E3-5A.2) showed significant phenotypic effects and represented GWAS-significant loci under both control and salt-stress conditions. Gene ontology analysis of the genomic regions linked to these QTNs revealed 67 putative candidate genes associated with ion transport, stress signaling, and photosynthetic processes. The identified SNPs, QTNs, and candidate genes provide valuable genomic resources for marker-assisted breeding of salt-tolerant wheat cultivars, contributing to sustainable wheat production under saline environments.

## 1 Introduction

Wheat (*Triticum aestivum* L.) is a valuable source of carbohydrates, proteins, and fibers for about nearly one-third of the world’s population (Grote et al., 2021). The global food requirement is increasing rapidly and is anticipated to double by 2050 (Nouraei et al., 2024). The rapidly growth of the global population, coupled with challenges posed by climate change underscores the urgent need for sustainable wheat production to safeguard food and nutritional security (Hu et al., 2021). Climate change is likely to exacerbate soil salinity in many regions, driven by rising sea levels that cause saltwater intrusion or the overuse of groundwater. In addition, the extent of saline land continues to expand annually because of factors like reduced rainfall, high evaporation rates, irrigation with saline water, and inadequate agricultural practices (Singh et al., 2018). Around 20% of global arable land and 50% of irrigated land experience reduced crop yields due to the negative effects of salt stress (Javid et al., 2025). Under saline conditions, plants experience marked declines in growth rate, accelerated leaf senescence, and reduced tillering; prolonged exposure further impairs reproductive development and ultimately leads to substantial declines in grain yield (Javid et al., 2022). Excessive salt significantly impacts wheat by destabilizing cellular membranes, diminishing chlorophyll synthesis, and restricting early plant development (Munns and Tester, 2008). Salinity stress causes the excessive damage during the vegetative and early reproductive growth stages (Chaurasia et al., 2020). Therefore, understanding the genetic basis of salt adaptation in wheat is critical for breeding initiatives aimed at developing salt-resilient varieties.

Salt tolerance is a complex trait involving numerous genes, signaling pathways, regulatory networks, and metabolic processes (Oyiga et al., 2018; Sun et al., 2018; Javid et al., 2022). Several physiological mechanisms have been linked with salt tolerance in wheat, including Na⁺ exclusion, compatible osmolyte accumulation, vacuolar compartmentalization of Na⁺, and regulation of a nominal Na⁺/K⁺ balance in leaves (Munns and Tester, 2008). The effectiveness of these tolerance mechanisms largely relies on a plant’s genetic and physiological responses to surrounding environmental factors (Quamruzzaman et al., 2022). The severity and duration of salt stress, along with genotypic and developmental differences, significantly influence the salinity response of wheat (Javid et al., 2022). Due to these complexities, screening of wheat genotypes for salt tolerance at the seedling growth stage and developing salt-tolerant wheat varieties remains a complex job for researchers. To improve salinity tolerance in wheat, it is crucial to investigate insights into the genetic and molecular architecture of associated traits for targeted breeding efforts. Conventionally, breeders have applied QTL mapping approach to elucidate the genetic basis of salinity tolerance in wheat (Dubcovsky et al., 1996; Guo et al., 2022). A prominent example is the salt tolerance locus *Nax2*, identified from a salt-tolerant *Triticum monococcum* genotype, which effectively conferred tolerance when introgressed into salt-susceptible durum wheat backgrounds (Munns et al., 2012). Several subsequent findings have identified QTLs associated with salinity tolerance across different wheat chromosomes using mapping populations derived from salt-tolerant parental crosses (Byrt et al., 2014; Turki et al., 2023; Khakshoor et al., 2025). However, despite these advances, the successful application of marker-assisted selection (MAS) for improving salinity tolerance in wheat remains limited, primarily due to the low resolution of traditional QTL mapping.

Genome-wide association study (GWAS) is widely recognized as an effective high-resolution approach for mapping genetic loci underlying complex plant traits. This strategy exploits allelic variation present in natural populations to precisely pinpoint trait-related loci (Chaurasia et al., 2020). GWAS studies have been carried out across several important crops including *Oryza sativa* L (Lu et al., 2025), *Zea mays* L (Oder et al., 2025), *Hordeum vulgare* L (Borrego-Benjumea et al., 2021), *Vigna radiata* (L.) R. Wilczek (Sinha et al., 2023), *Eleusine coracana* (L.) Gaertn (Kannababu et al., 2025), *Cicer arietinum* L (Raiya et al., 2025), *Glycine max* L (Bhat et al., 2022), *Brassica juncea* (L.) Czern. and Coss (Patel et al., 2025), as well as wheat to unravel the genetic basis of numerous agronomic and adaptive traits (Kumari et al., 2023; Nouraei et al., 2024; Atsbeha et al., 2025; Li et al., 2025a). Over the past few years, considerable efforts have been directed toward improving refining GWAS models to improve the genetic architecture of both simple and complex traits in plants. These models are broadly categorized as single-locus GWAS (SL-GWAS) and multi-locus GWAS (ML-GWAS) methods. Although single-locus GWAS methods have been extensively applied to identify genetic variants linked to several traits, a major limitation is the requirement for stringent multiple-testing corrections of marker *p*-values to control false-positive associations (Kumari et al., 2023). To address this limitation multilocus random-SNP-effect MLM (mrMLM) package was developed. The mrMLM package integrates six ML-GWAS methods, namely mrMLM (Wang et al., 2016b), FASTmrMLM (Tamba et al., 2017), pLARmEB (Zhang et al., 2017), pKWmEB (Ren et al., 2018), FASTmrEMMA and ISIS EM-BLASSO (Wen et al., 2018). Collectively, these multilocus GWAS models improve detection power and mapping accuracy by simultaneously considering multiple loci, thereby providing a more reliable framework for elucidating the genetic basis of complex traits.

The present study was carried out with the aim to dissect the genetic and molecular mechanisms of salinity tolerance in wheat with a focus on discovering beneficial novel alleles from germplasm resources for strengthening breeding efforts. Multi-locus GWAS approach was applied on a diverse set of wheat genotypes to uncover novel genomic regions linked to salt tolerance. The findings of this study are expected to provide insights into potential mechanisms behind salt tolerance during the vegetative phase in wheat and to facilitate marker-assisted breeding for developing improved salinity tolerant varieties of wheat.

## 2 Materials and methods

### 2.1 Plant material and growth conditions

The plant material comprised of 313 diverse accessions of bread wheat (**Supplementary Table S1**) along with 4 check genotypes with two tolerant (KRL-210, Kharchia-65: K-65), and two sensitive (HD2851, and HD3226) genotypes. The seeds of these genotypes were procured from the Indian National Gene Bank, ICAR-NBPGR, New Delhi. The screening study was performed under controlled conditions at the National Phytotron Facility, IARI, New Delhi. The hydroponic screening experiment was conducted for two consecutive years 2021 and 2022. Seedlings were grown under a salinity treatment of 15 dS m^-1^ consisting of a mixture of 100 mM of NaCl, 18.60 mM of CaCl_2_·2H_2_O, and 25.04 mM of Na_2_SO_4_, which creates 4:1 of Na^+^:Ca^2+^ and Cl^-^:SO_4_^2-^, respectively, along with control group (Boopalet al., 2023). Surface sterilization of wheat seeds was carried out using 2% sodium hypochlorite (NaClO), and the seeds were subsequently washed four to five times with deionized water to remove remaining traces. The surface-sterilized seeds were subsequently placed in wet cellulose germination paper for germination. After seven days, healthy and uniformly grown seedlings were selected for transplantation, fixed in acrylic sheets with foam supports, and placed in plastic trays containing 20 L of modified Hoagland nutrient solution (**Supplementary Figure S1**) (Boopal et al., 2023). Each acrylic sheet consisted of 90 holes, three seedlings were planted per hole and, each genotype was arranged in three replications in a tray of control and salt stress. Total 90 genotypes were transplanted per tray amounting to 12 trays for each control and salt stress with checks planted after 20 genotypes. The Hoagland nutrient solution was refreshed every 3–4 days, and the pH was adjusted to 5.2-5.6 using 0.1 M HCl (acid regulator) and 0.1 M NaOH (base regulator). The pH was monitored and maintained throughout the experiment using a portable pH meter (Waterproof Pocket pH Tester with 0.1 resolution - pHep^®^ HI 98107, Hanna Instruments, Inc., USA). The altered Hoagland nutritional solution was formulated in deionized reverse osmosis water. The salinity and electrical conductivity were calibrated via a portable EC meter (HI 8733, Hanna Instruments, Inc., USA). The day and night times temperatures were maintained at 22 °C and 12 °C, respectively. Plants were grown under a 10h light/12h dark photoperiod at a photon flux density of 400 μmol m⁻^2^ s^-1^, and glasshouse relative humidity varied from 80% to 90%. All measurements and samplings were conducted on 30-day old plants after 23 days of growth under both salinity treatment and control conditions.

### 2.2 Leaf chlorophyll content index

Chlorophyll content of leaves from both control (CCI-C1: control 2021, CCI-C2: control 2022) and salt-treated (CCI-T1: treatment 2021, CCI-T2: treatment 2022) plants was measured in thirty-days-old seedlings after 23 days growth under salinity and control conditions using the CCM-200 Plus chlorophyll content meter (Opti-Sciences Inc., USA). The readings were taken from the uppermost complete expanded portion of the terminal leaves of plants per replicate for each treatment (Boopal et al., 2023).

### 2.3 Green leaf area

The area of all green and photosynthetically active leaves was measured using a LiCOR 3100 leaf area meter (LI-COR Environmental, USA). Leaves from thirty-day-old seedlings after 23 days growth under salinity (GLA-T1: treatment 2021, GLA-T2: treatment 2022) and control (GLA-C1: control 2021, GLA-C2: control 2022) conditions were collected from replicated trays, placed in butter paper bags, and stored in an ice bucket for a short period until measurement.

### 2.4 Dry biomass

Plants from replicated trays were harvested and their dry weight was measured using a weighing scale. Before weighing, plants were dried in an oven until a stable weight was attained, after which their dry biomass (DB) was measured (Boopal et al., 2023) for both control (DB-C1: control 2021, DB-C2: control 2022) and salinity stress (DB-T1: treatment 2021, DB-T2: treatment 2022).

### 2.5 Phenotypic data analysis

Descriptive statistics for all traits under control and salinity stress were computed using SPSS statistical software (IBM Corp, 2020). Pearson’s correlation between different years for the same traits, as well as within the same year for different traits was calculated using the ‘metan’ R-package (Olivoto and Lúcio, 2020). Principal component analysis (PCA) and hierarchical cluster analysis was analysed using the factoextra (Kassambara and Mundt, 2020) and plotted with ‘ggplot2’ (Wickham, 2017) packages in R. (R Core Team, 2023) BLUP values were calculated using META-R (Alvarado et al., 2020) for individual years as well as for combined across year 2021 and 2022.

### 2.6 DNA isolation and SNP genotyping

The MACHEREY-NAGEL NucleoSpin^®^ Plant II Mini Kit was used to isolate genomic DNA from 15-day-old seedlings, following the manufacturer’s standard protocol. DNA quality was checked on a 0.8% agarose gel, and DNA concentration was quantified using a NanoDrop 1000 spectrophotometer (Thermo Scientific). SNP genotyping was carried out using the Breeders’ 35K Axiom^®^ array according to the Affymetrix protocol (Allen et al., 2017). SNPs with a call rate below 90% and a minor allele frequency (MAF) below 10% were excluded from further analysis.

### 2.7 Population structure and linkage disequilibrium analyses

A Bayesian model-based method available in the STRUCTURE software was used to analyze population structure (Pritchard et al., 2000). The number of assumed subpopulations (K) was set to range from 1 to 10, with 25,000 burn-in iterations and 50,000 MCMC replications. The ideal number of subpopulations was determined based on the maximum ΔK value (Evanno et al., 2005) using StructureSelector web (Li and Liu, 2017). Linkage disequilibrium (LD) decay was estimated for each wheat subgenome (A, B, and D) as well as for the whole genome using TASSEL v5.0 (Bradbury et al., 2007). The neighbor-joining (NJ) approach in Genome Association and Prediction Integrated Tool (GAPIT3) was used to generate a genotypic dendrogram of 313 wheat genotypes (Wang and Zhang, 2021).

### 2.8 GWAS analysis

Genome-wide association analysis was estimated through the mrMLM package v.4.0 (https://cran.r-project.org/package=mrMLM) in R. Six ML-GWAS models: mrMLM, FASTmrEMMA, FASTmrMLM, ISIS EM-BLASSO, pLARmEB, and pKWmEB were used with default parameters. QTNs showing significant association with salt tolerance were defined based on a LOD score threshold of ≥3.00. Additionally, SNP markers identified by a minimum of two different models were designated as reliable QTNs for salt tolerance.

### 2.9 Detection of candidate genes and *in-silico* expression

To identify candidate genes or transcripts related to salt tolerance, probe sequences of SNPs significantly identified through GWAS were aligned to the wheat reference genome (IWGSC RefSeq v1.0). The alignments were performed using the BLAST tool with default settings in the Ensembl Plants database (https://plants.ensembl.org/Triticum_aestivum/Tools/Blast). Potential candidate genes were identified as those located within LD upstream and downstream of the candidate genes. To investigate the expression of these putative candidate genes, an *in-silico* analysis was conducted using RNA-seq data (NCBI Bioproject: PRJNA487922) (Amirbakhtiar et al., 2021) of wheat leaves under control and salt stress conditions. The dataset was re-analysed in the present study as described below. Data integrity was ensured by FastQC v0.12.1 (Andrews, 2010) and low-quality sequences, adapter sequences were trimmed using Trimmomatic v0.39 (Bolger et al., 2014). The reference genome (RefSeq v2.1) of cv. Chinese Spring (CS) and corresponding gene annotation files for *Triticum aestivum* were sourced from the International Wheat Genome Sequencing Consortium (IWGSC) (https://wheatgenome.org/projects/reference-genome-project/refseq-v2.1) (Zhu et al., 2021). The STAR alignment tool (v2.7.11b) (Dobin and Gingeras, 2015) was used to align the clean reads to the reference genome. SAMtools v1.22.1 (Li et al., 2009) was used for sorting and indexing. Gene quantification was performed using featureCounts v2.0.0 (Liao et al., 2014) to generate read counts from the aligned BAM files. A heatmap was constructed from the FPKM (Fragments Per Kilobase of Transcript per Million mapped reads) values of the candidate genes using the pheatmap R package (Kolde, 2019). Additionally, the spatio-temporal expression patterns of the candidate genes were examined using the Wheat Expression Browser (https://www.wheat-expression.com/).

### 2.10 Significant QTN genotype-phenotype association analysis

To assess the phenotypic impact of GWAS identified QTNs, a post-GWAS analysis was conducted using parametric or non-parametric statistical frameworks using methodology described by Nawade et al. (2025). Genotype effects were tested by ANCOVA with genotypes as fixed and PC1-PC3 as covariates, model assumptions were tested using Shapiro-wilk for normality and Levene’s test for homogeneity. When ANCOVA assumptions were violated, resulting residuals were analysed with Kruskal-Wallis by adjusting population structure. Allelic effects were quantified using ∆Mean (Nawade et al., 2025).

## 3 Results

### 3.1 Variation in phenotypic traits

Descriptive statistics for measured traits chlorophyll content index (CCI), green leaf area (GLA), and dry biomass (DB) under control (C1- Control 2021; C2- control 2022) and salinity stress (T1- salinity treatment 2021; T2- salinity treatment 2022) in two years indicated clear differences in magnitude and variability among traits. Under control conditions, mean CCI were identical in C1 and C2 (3.75), with moderate variability (Standard Deviation-SD 1.07-1.11), while GLA with mean of ~39 showed highest dispersion (SD 18.84-19.52) and positively skewed distributions reflecting substantial genotypic variation. DB under control conditions exhibited lower mean values 0.36 with relatively small variance, although the distributions were positively skewed and leptokurtic, suggesting the presence of genotypes with comparatively higher biomass. Under salt stress conditions, mean value for all traits declined markedly in comparison to controls particularly for CCI (~1.99) and GLA (~12.8), indicating adverse effect of stress on photosynthetic ability. GLA-T1 and GLA-T2 declined by 67.70% and 67.08%, respectively. Similarly, CCI-T1 and CCI-T2 decreased by 46.93% and 47.20%, respectively. Similar effects of salt stress were detected on DB-T1 and DB-T2 decreased by 44.44% and 47.22%, respectively, compared to the control. GLA and DB exhibited comparatively higher coefficient of variation (CV) indicating substantial variability under both stress and control conditions. In contrast, CCI showed relatively low variability, with lesser CV values reflecting greater trait stability irrespective of environmental conditions (**Table 1**). Additionally, we compared the performance of genotypes under salt stress with the tolerant checks (K65 and KRL210) which led to the identification of several genotypes exhibiting superior performance. For CCI, genotypes IC111914, IC0443766, IC384555, IC539415 and IC573155 (range 4.33-3.33) exhibited markedly higher values than both K65 (3.08) and KRL210 (2.64). In terms of DB IC335977, IC532880, IC384555 and IC128386 (0.57-0.51) surpassed K65 (0.50). Similarly, for GLA IC356111, IC395828 and IC532880 (39.10-35.14) performed better than KRL210 (35.14). Interestingly, IC532880 displayed consistent superiority across DB and GLA, while IC384555 also performed well for CCI and DB indicating their potential as salt-tolerant genotypes.

**Table 1:** Evaluation of salinity tolerance traits in wheat association panel.

Traits^#^	Treatments	Min	Max	Mean	SE	SD	CV (%)
CCI-C1	Control	1.50	7.05	3.75	0.06	1.11	29.60
CCI-T1	Treatment (Salt)	0.00	4.25	1.99	0.03	0.53	26.63
CCI-C2	Control	1.63	8.53	3.75	0.06	1.07	28.53
CCI-T2	Treatment (Salt)	0.00	4.42	1.98	0.03	0.53	26.76
GLA-C1	Control	8.39	117.89	39.66	1.04	19.52	49.21
GLA-T1	Treatment (Salt)	0.00	42.79	12.81	0.47	8.88	69.32
GLA-C2	Control	7.56	110.59	39.04	1.01	18.84	48.25
GLA-T2	Treatment (Salt)	0.00	45.91	12.85	0.47	8.79	68.40
DB-C1	Control	0.10	1.03	0.36	0.01	0.17	47.22
DB-T1	Treatment (Salt)	0.05	0.62	0.20	0.01	0.10	50.00
DB-C2	Control	0.11	1.11	0.36	0.01	0.19	52.77
DB-T2	Treatment (Salt)	0.05	0.53	0.19	0.01	0.10	52.63

**^#^**Where, CCI-T1, chlorophyll content index-2021; CCI-T2, chlorophyll content index-2022; GLA-T1, green leaf area-2021; GLA-T2, green leaf area-2022; DB-T1, Dry biomass-2021; DB-T2, dry biomass-2022. C and T represent the control and treatment conditions, respectively.

### 3.2 Pearson’s correlation among phenotypic traits

**Figure 1:**
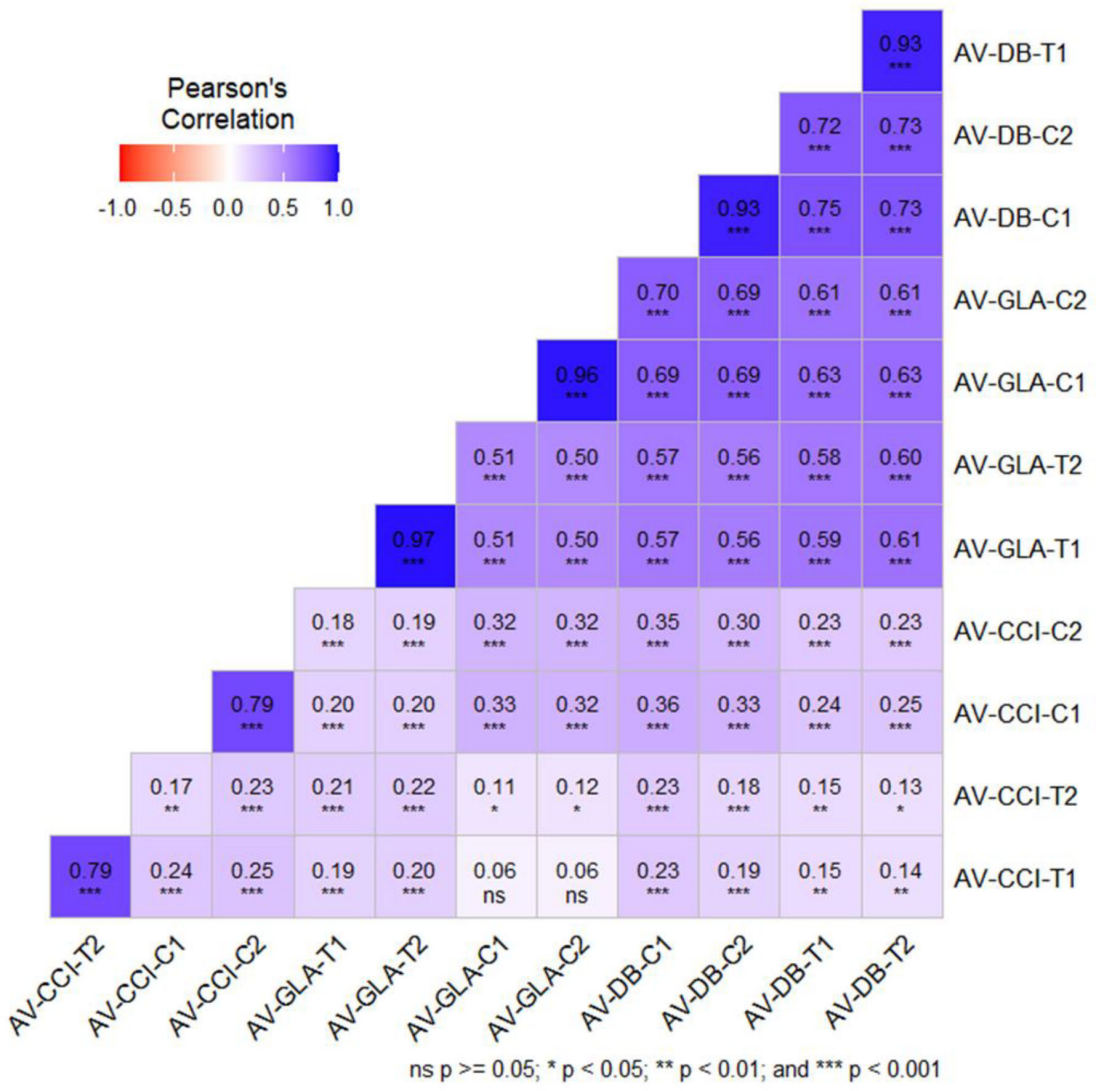
Pearson’s correlation bar plot between different phenotypic traits of 313 wheat genotypes. ns, p > 0.05; *p < 0.05; **p < 0.01; ***p < 0.001.

Pearson’s correlation analysis revealed consistent and positive relationships among CCI, GLA and DB under both control and stress conditions across years. Strong and highly significant positive correlations were observed among GLA and DB (r: 0.56-0.70, ***p < 0.001) across control and stress environments indicating that genotypes with larger leaf area tends to accumulate higher biomass irrespective of conditions. Under control conditions CCI exhibited a moderately positive correlation with GLA and DB (GLA-C1: r = 0.327; GLA-C2: r = 0.317 and DB-C1: r = 0.356; DB-C2: r = 0.299), suggesting that greater biomass and leaf area were linked to higher chlorophyll content. However, under salt stress, there weak positive correlations were observed between CCI with GLA-T1 (r = 0.186), GLA-T2 (r = 0.215), DB-T1 (r = 0.149) and DB-T2 (r = 0.136). These findings imply that although chlorophyll content has a positive correlation with biomass and leaf area in all circumstances, the strength of these correlations decreases under treatment stress, suggesting that the physiological link between traits may be disrupted in less-than-ideal growing environments. Additionally, strong correlations were observed for all traits, in particular DB showed high correlations with r ~ 0.93-0.97 reflecting strong trait stability across years (**Figure 1**).

### 3.3 Principal component analysis

**Figure 2:**
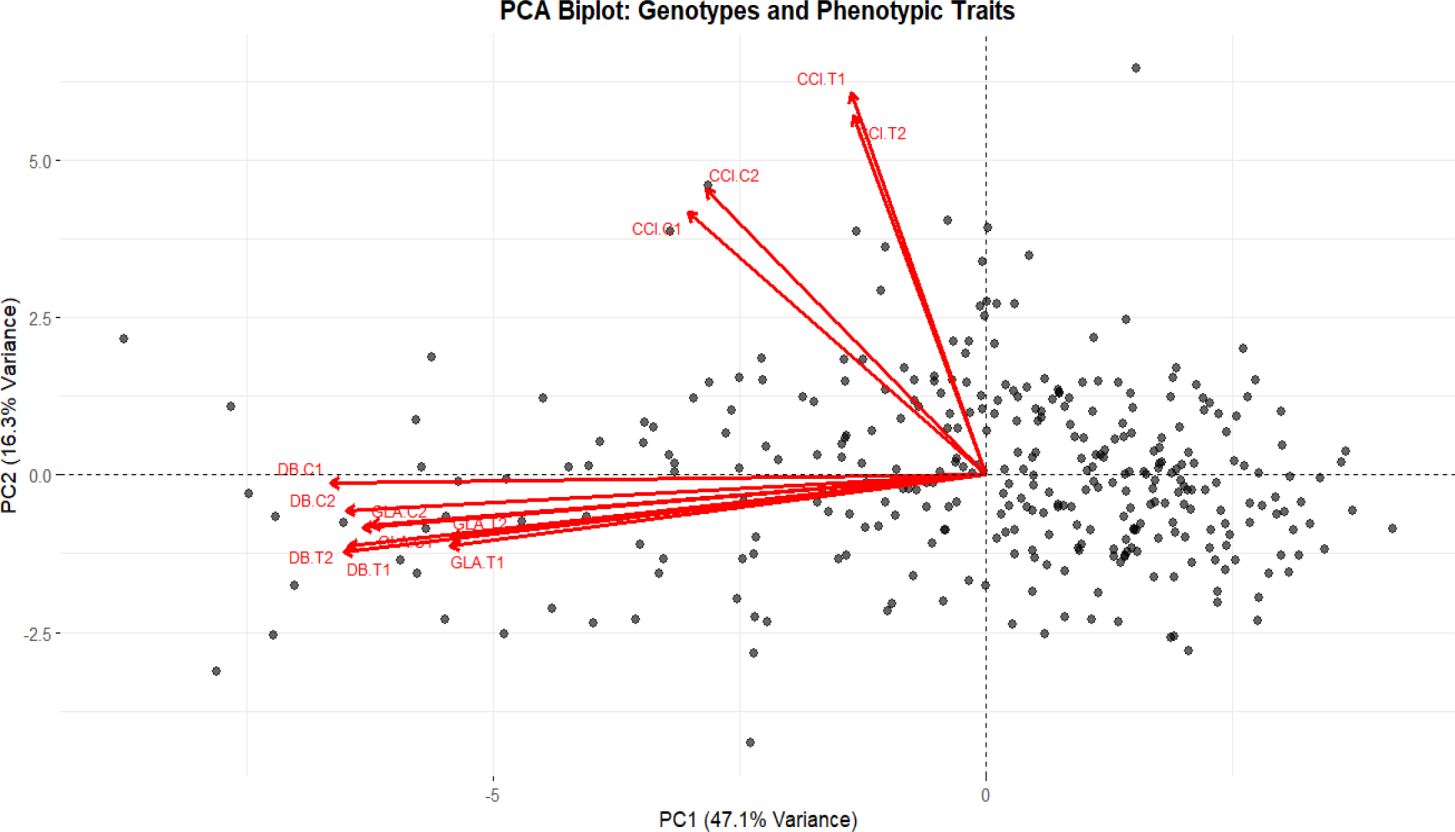
Biplot showing the principal component analysis (PCA) of phenotypic traits in wheat.

The Principal Component Analysis (PCA) under control and salt stress conditions revealed that the first two principal components explained a substantial proportion of the total phenotypic variation with principal component (PC1), accounting for 47.1% of the variance and PC2 for 16.3%, together capturing 63.4% of the overall variability. High contribution of PC1 indicated that majority of phenotypic difference among genotypes was driven by growth related traits, namely DB and GLA which clustered together and exhibited a strong positive correlation under both control and stress conditions as previously indicated by Pearson’s correlation. On the other hand, PC2 was mainly influenced by CCI traits indicating that variation in CCI contributed independently to total phenotypic variance. Overall, the PCA biplot highlights that GLA and DB traits have a positive correlation with one another but a negative correlation with CCI trait, indicating that different genotypes have different physiological reactions to various circumstances (**Figure 2**).

### 3.4 SNPs genotyping, linkage distribution and population structure

A total of 24,968 high-quality SNPs were used for genotyping, of which 7,785 were distributed on sub-genome A, 9,396 on sub-genome B, and 7,787 on sub-genome D. The distribution of SNP markers per chromosomes varied from 486 (4D) to 1,788 (1B). In sub-genome A, SNPs were most abundant on chromosome 2A (1,308), with slightly lower numbers on chromosomes 1A (1,253) and 7A (1,219). Sub-genome B showed the greatest SNP abundance on chromosome 1B (1,788), followed by chromosomes 2B (1,615) and 5B (1,440). Similarly, in sub-genome D, chromosome 2D carried the highest number of SNPs (1,617), followed by 1D (1,554) and 3D (1,140). Among all chromosomes, 4D exhibited the maximum SNP density (1.049 SNPs/Mb), while chromosome 1D showed the minimum density (0.318 SNPs/Mb) (**Table 2**).

**Table 2:** Distribution of SNPs and intra-chromosomal LD across wheat chromosomes in 313 genotypes.

S. No.	Chr	Size (Mb)	No. of SNP	SNP/Mb Density	Average LD (r2)	D prime
1	1A	594.1	1253	0.474	0.164	0.567
2	1B	689.85	1788	0.385	0.210	0.613
3	1D	495.45	1554	0.318	0.205	0.572
4	2A	780.8	1308	0.596	0.144	0.497
5	2B	801.26	1615	0.496	0.167	0.558
6	2D	651.85	1617	0.403	0.181	0.542
7	3A	750.84	1088	0.690	0.168	0.565
8	3B	830.83	1274	0.652	0.178	0.576
9	3D	615.55	1140	0.539	0.110	0.453
10	4A	744.59	802	0.928	0.142	0.507
11	4B	673.62	726	0.927	0.156	0.509
12	4D	509.86	486	1.049	0.135	0.460
13	5A	709.77	1187	0.597	0.182	0.570
14	5B	713.15	1440	0.495	0.202	0.606
15	5D	566.08	1103	0.513	0.218	0.557
16	6A	618.08	928	0.666	0.149	0.546
17	6B	720.99	1323	0.544	0.148	0.543
18	6D	473.59	851	0.556	0.095	0.423
19	7A	736.71	1219	0.604	0.144	0.552
20	7B	750.62	1230	0.610	0.138	0.514
21	7D	638.69	1036	0.616	0.089	0.426

**Figure 3:**
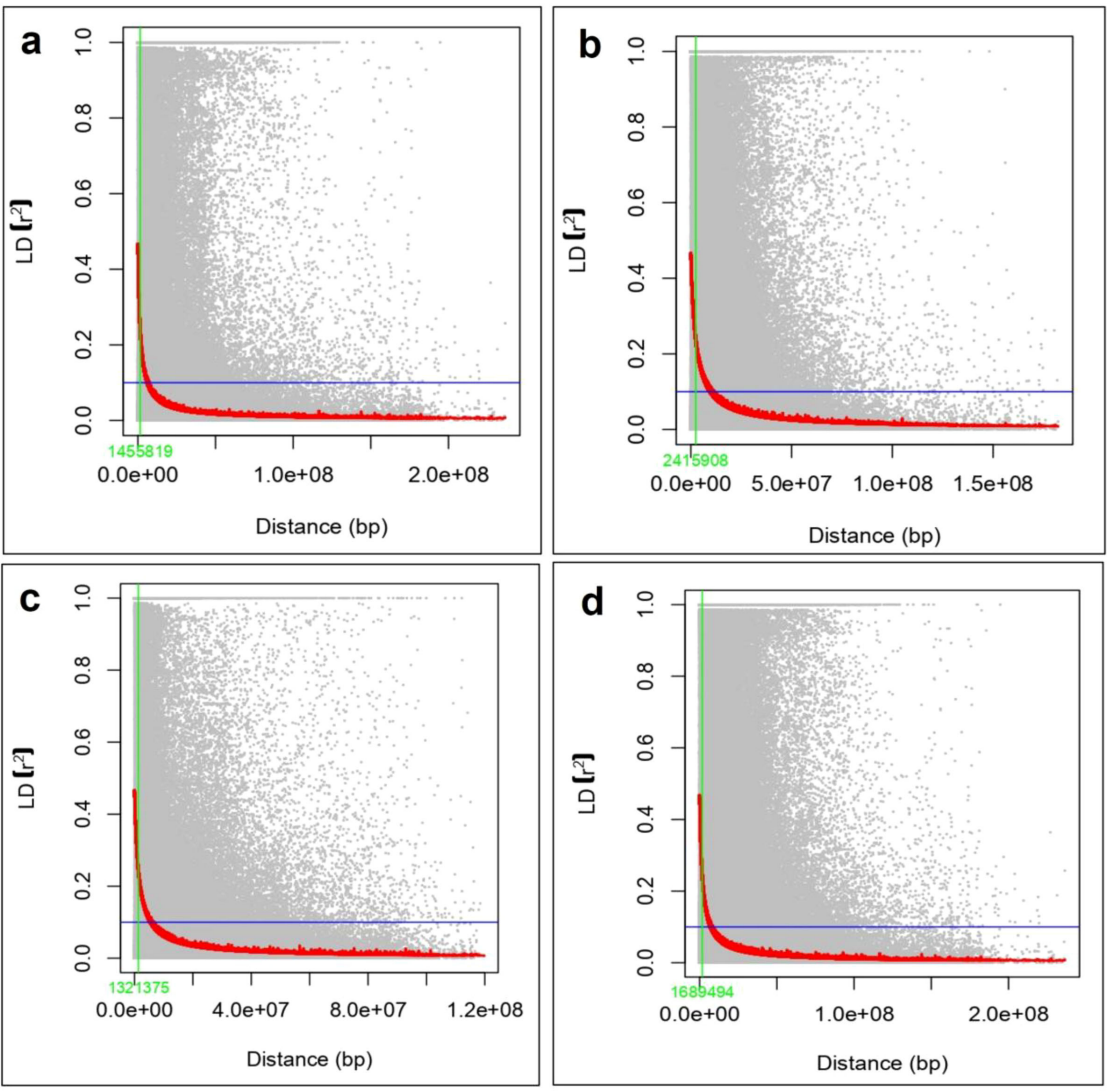
Linkage disequilibrium (LD) of; **(a)** A genome, **(b)** B genome, **(c)** D genome, **(d)** whole genome in wheat.

In addition, genome-wide LD was conducted for whole genome as well as for sub-genomes A, B and D in the wheat AM panel was identified using 24,968 SNPs markers. Among the sub-genomes, B exhibited lowest LD decay of 2.42 Mb while LD decay was most rapid in D at 1.32 Mb. The A sub-genome showed LD decay at intermediate level at 1.45 Mb. For the whole genome LD decay was observed at 1.69 Mb (**Figure 3**). Among the 21 wheat chromosomes, LD (r^2^) ranged between 0.089 (Chr7D) to 0.210 (Chr1B). Among these, the highest number of SNP pairs (1,788) were observed on chromosome 1B, while the lowest number of SNPs pairs (148) were observed on chromosome 4D. A maximum and minimum value of D prime was found in 1B (0.613) and 6D (0.423), respectively.

**Figure 4:**
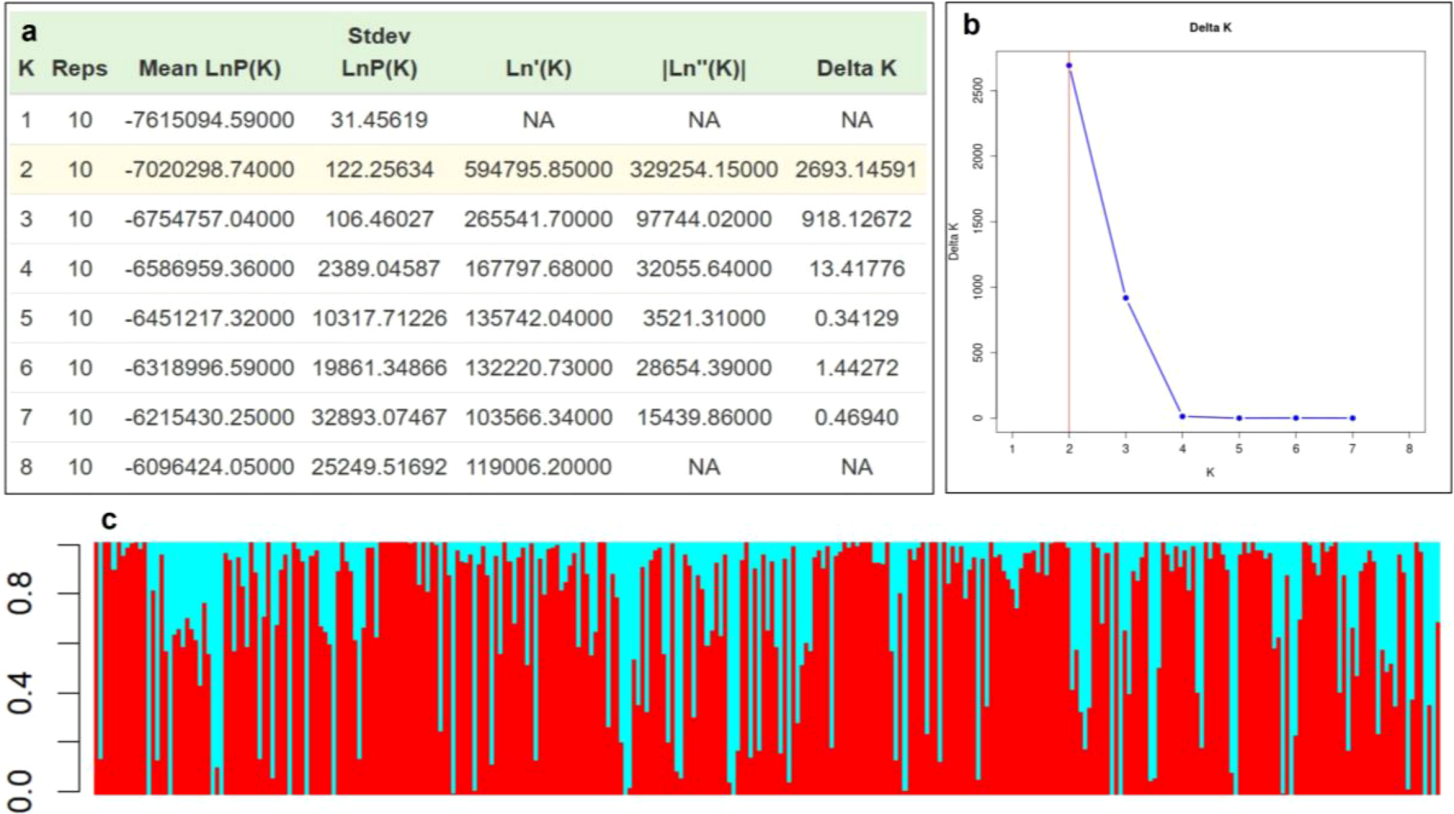
The population structure of the 313 AM panel, **(a)** delta K value is maximum at 2, **(b)** bar plot of delta K (K = 2), **(c)** two sub-population groups of 313 genotypes from sub-population I and sub-population II shown in red and cyan colour, respectively.

Bayesian clustering analysis partitioned the 313 genotypes into two genetic groups (K = 2) (**Figures 4a, b**). Sub-population I comprised 12.77% pure lines and 87.23% admixed genotypes, whereas sub-population II contained only 4.79% pure and 95.20% admixed individuals, indicating substantial historical recombination and gene flow. All exotic accessions from Mexico, Australia, Nepal, and the USA clustered in sub-population I, except three USA genotypes (EC38113, EC609338, and EC6903). Indigenous genotypes from Uttarakhand, Himachal Pradesh, Haryana, and Punjab were distributed across both groups (**Figure 4c**), reflecting shared ancestry and genetic exchange between local and exotic germplasm.

**Figure 5:**
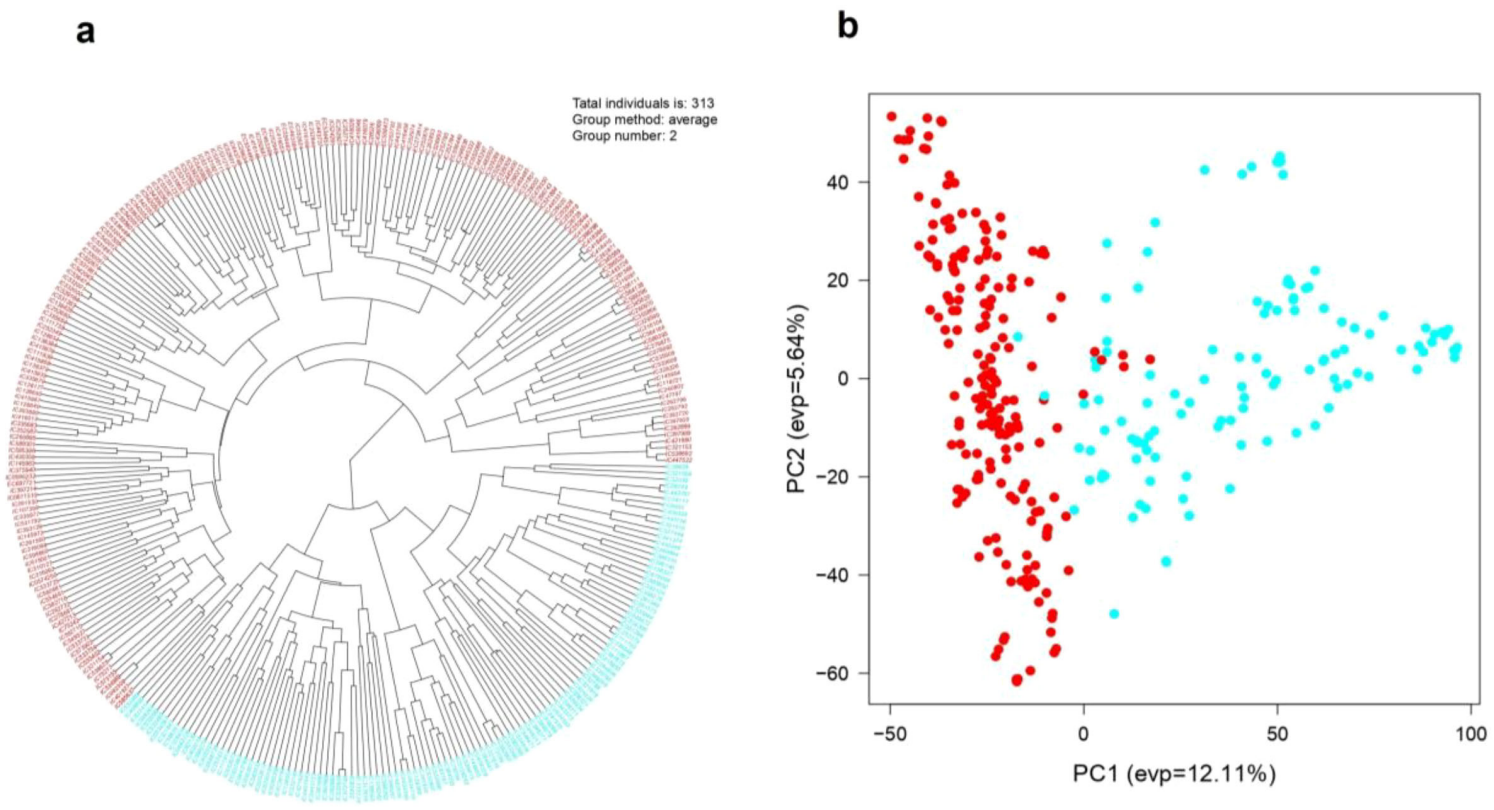
**(a)** NJ tree, and **(b)** principal component analysis (PCA) showing the two major sub-populations in 313 wheat genotypes.

The Neighbor-Joining tree and principal component analysis (PCA) also revealed similar and consistent clustering patterns among the genotypes (**Figures 5a, b**). Principal component analysis (PCA) of the AM panel revealed that PC1 and PC2 contributed 12.11% and 5.64% of the total genetic variance, respectively (**Figure 5b**) and clearly separated the genotypes into two major groups. Similarly, the Neighbor-Joining (NJ) dendrogram constructed for 313 wheat genotypes also grouped the panel in two major clusters (Group 1 and Group 2) implying the presence of two major lineages (**Figure 5a**).

### 3.5 Genome-wide association analysis

A total of 68 significant QTNs linked to three salt tolerance traits were detected through six multi-locus GWAS approaches in the mrMLM package, applying a LOD threshold of ≥ 3. Among these, 25 and 19 QTNs were identified under control and treatment conditions, respectively (**Supplementary Tables S2**, **S3**). Each QTN explained between 1.47E-07% and 23.89% of the phenotypic variation (R^2^). These QTNs were distributed across 21 chromosomes in the A, B, and D sub-genomes, with the highest numbers of combined QTNs located on chromosomes 1A, 3B, and 4A, followed by 1B, 4D, 5A, 5D, 7B, 2B, 3A, 4B, 6B, and 7A.

**Figure 6:**
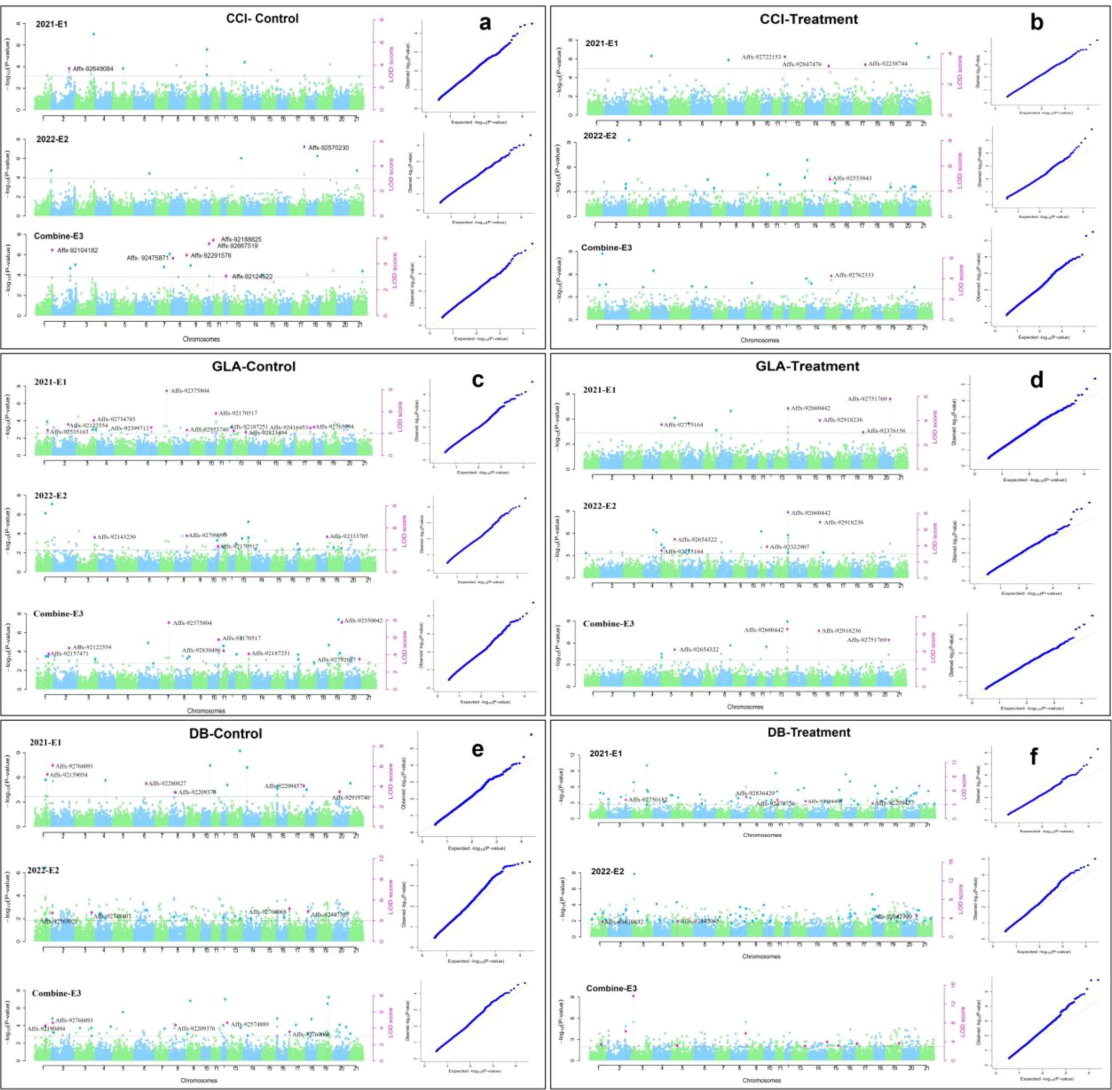
Manhattan plots displaying QTNs significantly linked to three traits: **(a)** CCI-control, **(b)** CCI-treatment, **(c)** GLA-control, **(d)** GLA-treatment, **(e)** DB-control, **(f)** DB-treatment identified using six different multi-locus GWAS models in 313 genotypes of wheat. The horizontal lines indicate the significance threshold (LOD score = 3).

Among the total 68 identified QTNs associated with three different traits, 24 QTNs were repeatedly identified across at least two models. These combined QTNs were considered robust and reliably linked to their respective traits (**Table 3**). Overall, the 68 QTNs were distributed among the traits as follows: CCI, GLA, and DB (**Figure 6**).

**Table 3:** Twenty-four salt tolerance QTNs identified in the combined analysis using at least two multi-locus GWAS models.

Trait	QTN	Marker	Marker position (bp)	Allele	LOD score	‘-log10(P)’	r2 (%)	Method
Chlorophyll content index (CCI-E3)	Q.CCI-E3-1A	Affx-92104182	1A:590531935	A/G	3.47-5.70	4.19- 6.53	2.80- 8.04	1, 5, 2, 4, 6
	Q.CCI-E3-3B.1	Affx-92475871	3B:20074481	G/T	4.43-4.71	5.45- 5.49	2.27- 4.00	1, 2, 6
	Q.CCI-E3-3B.2	Affx-92291576	3B:787721940	C/A	4.67	5.45	5.97- 7.20	1, 2
	Q.CCI-E3-4A.1	Affx-92667519	4A:602797969	T/C	5.55	6.37	9.12- 9.51	1, 2
	Q.CCI-E3-4A.2	Affx-92188825	4A:735067552	T/C	4.10- 5.82	4.85- 6.64	7.46- 16.79	1, 2, 6
	Q.CCI-E3-4D	Affx-92124522	4D:145507672	T/G	3.06	3.76	1.93-4.13	1, 2
	Q.CCI-E3-5D	Affx-92553843	5D:334697005	C/A	3.36- 5.14	4.08- 5.9	10.58- 11.20	4, 1
Green leaf area (GLA- E3)	Q.GLA-E3-1A	Affx-92157471	1A:503993416	C/T	4.06- 4.14	4.82- 4.90	7.33- 14.49	4, 6
	Q.GLA-E3-1B	Affx-92122554	1B:492301285	G/A	4.26- 5.26	5.02- 6.0	3.16- 6.46	3, 4
	Q.GLA-E3-3A	Affx-92375804	3A:538639421	C/T	3.54- 11.10	4.27- 12.06	2.84-9.85	1, 2, 6
	Q.GLA-E3-4A	Affx-92170517	4A:737096657	G/A	4.92- 6.88	5.71- 7.74	4.88- 9.47	2, 3, 4, 6
	Q.GLA-E3-4B	Affx-92830496	4B:424749871	C/T	3.45- 5.46	4.17- 6.27	8.24-10.24	5, 4
	Q.GLA-E3-5A.1	Affx-92187251	5A:610217061	G/A	3.42- 4.78	4.14- 5.57	1.56-3.02	2, 6
	Q.GLA-E3-7A	Affx-92350042	7A:658856557	G/A	5.58- 12.12	6.39- 13.10	5.95- 14.07	3, 5, 6
	Q.GLA-E3-7B.1	Affx-92792697	7B:708913755	T/C	3.48-3.51	4.20- 4.24	1.68- 2.21	2, 6
	Q.GLA-E3-2B	Affx-92654322	2B:667805353	G/A	3.20- 4.22	3.90- 4.98	9.76- 13.31	4, 6
	Q.GLA-E3-5A.2	Affx-92660442	5A:673405475	T/G	5.69- 8.58	6.51- 9.48	10.14- 13.46	2, 5, 4, 6
	Q.GLA-E3-5D	Affx-92918236	5D:537222235	C/T	3.83- 9.02	4.57- 9.93	6.48- 12.36	1, 2, 3, 5
	Q.GLA-E3-7B.2	Affx-92751769	7B:670443615	C/T	4.03- 6.38	4.79- 7.23	6.95- 9.47	4, 6
Dry biomass (DB-E3)	Q.DB-E3-1A	Affx-92190494	1A:471071111	T/C	3.35- 8.08	4.07- 8.98	3.86-18.02	1, 3, 4, 6
	Q.DB-E3-1B	Affx-92766091	1B:14030253	C/T	4.11- 12.81	4.87- 13.80	7.64- 16.53	1, 2, 4, 6
	Q.DB-E3-3B	Affx-92209376	3B:278328740	C/G	3.61-5.11	4.34- 5.91	9.32- 12.47	1, 2
	Q.DB-E3-4D	Affx-92574889	4D:499503158	G/C	4.41- 4.95	5.18- 5.74	2.68- 3.97	1, 2
	Q.DB-E3-6B	Affx-92760068	6B:11540123	T/C	3.29- 3.84	4.01- 4.58	2.28- 2.77	3, 4

1, mrMLM; 2, FASTmrMLM; 3, FASTmrEMMA; 4, pKWmEB; 5, pLARmEB; 6, ISIS EM-BLASSO.

#### 3.5.1 QTNs associated with the chlorophyll content index trait

Seven QTNs significantly associated with the chlorophyll content index (CCI) trait were distributed across five chromosomes: 1A, 3B, 4A, 4D, and 5D. These QTNs such as Q.CCI-E3-1A, Q.CCI-E3-3B.1, Q.CCI-E3-3B.2, Q.CCI-E3-4A.1, Q.CCI-E3-4A.2, Q.CCI-E3-4D, and Q.CCI-E3-5D explained 2.80-8.04%, 2.27-4.00%, 5.97-7.20%, 9.12-9.51%, 7.46-16.79%, 1.93-4.13%, and 10.58-11.20% of the total phenotypic variation, respectively. Among them, Q.CCI-E3-5D contributed the maximum phenotypic variation (10.58-11.20%), while Q.CCI-E3-4D had the minimum (1.93-4.13%). Notably, Q.CCI-E3-4A.2 was the most prominent QTN, exhibiting the highest LOD score (4.10-5.82).

#### 3.5.2 QTNs associated with the green leaf area trait

Twelve QTNs significantly associated with the green leaf area (GLA) trait were distributed across 10 chromosomes: 1A, 1B, 2B, 3A, 4A, 4B, 5A, 5D, 7A, and 7B. These QTNs such as Q.GLA-E3-1A, Q.GLA-E3-1B, Q.GLA-E3-3A, Q.GLA-E3-4A, Q.GLA-E3-4B, Q.GLA-E3-5A.1, Q.GLA-E3-7A, Q.GLA-E3-7B.1, Q.GLA-E3-2B, Q.GLA-E3-5A.2, Q.GLA-E3-5D, and Q.GLA-E3-7B.2 explained 7.33-14.49%, 3.16-6.46%, 2.84-9.85%, 4.88-9.47%, 8.24-10.24%, 1.56-3.02%, 5.95-14.07%, 1.68-2.21%, 9.76-13.31%, 10.14-13.46%, 6.48-12.36%, and 6.95-9.47% of the total phenotypic variation, respectively. Among them, Q.GLA-E3-5A.2 contributed the maximum phenotypic variation (10.14-13.46%), while Q.GLA-E3-7B.1 contributed the minimum (1.68-2.21%). Notably, Q.GLA-E3-7A was the most prominent QTN, exhibiting the maximum LOD score (5.58-12.12).

#### 3.5.3 QTNs associated with the dry biomass trait

Five QTNs significantly associated with the DB trait were distributed across five chromosomes: 1A, 1B, 3B, 4D, and 6B. These QTNs including Q.DB-E3-1A, Q.DB-E3-1B, Q.DB-E3-3B, Q.DB-E3-4D, and Q.DB-E3-6B explained 3.86-18.02%, 7.64-16.53%, 9.32-12.47%, 2.68-3.97%, and 2.28-2.77% of the total phenotypic variation, respectively. Among them, Q.DB-E3-1A contributed the maximum phenotypic variation (3.86-18.02%), while Q.DB-E3-6B contributed the minimum (2.28-2.77%). Notably, Q.DB-E3-1B was the most prominent QTN, exhibiting the highest LOD score (4.11-12.81).

### 3.6 Phenotypic impact of GWAS-associated SNPs

To investigate whether QTNs detected by GWAS contributed to observable phenotypic variation, 24 SNPs detected in two or more GWAS models were subjected to genotype-phenotype comparisons. Significant genotype dependent phenotypic variation was observed across CCI, GLA and DB. Out of the 24 QTNs, 15 exhibited statistically significant differences with 9 QTNs being highly significant (p <0.0001). These highly significant QTNs included three associated with CCI (Q.CCI-E3-1A, Q.CCI-E3-4D, and Q.CCI-E3-5D), four with GLA (Q.GLA-E3-5A.1, Q.GLA-E3-5A.2, Q.GLA-E3-2B, and Q.GLA-E3-7B.2), and two with DB (Q.DB-E3-3B and Q.DB-E3-1B) (**Table 4**).

**Table 4:** Significant candidate QTNs from GWAS and their effects.

QTN	Marker	Allele (A/B)	Trait	Allele A	Allele AB	Allele B	Mean A	Mean AB	Mean B	Significance^#^	Delta mean
Q.CCI-E3-1A	Affx-92104182 (Exon: TraesCS1A02G445000)	A/G	CCI-Mean (Control)	157	86	57	3.48	3.7	4.21	***	0.73
Q.CCI-E3-4D	Affx-92124522 (Intergenic)	T/G	CCI-Mean (Control)	246	34	30	3.76	3.6	3.14	***	0.62
Q.CCI-E3-4A.1	Affx-92667519 (Exon: TraesCS4A02G309600)	T/C	CCI-Mean (Control)	195	68	38	3.50	3.8	4.31	**	0.81
Q.CCI-E3-3B.1	Affx-92475871 (Intergenic)	G/T	CCI-Mean (Control)	208	8	97	3.60	3.8	3.87	**	0.28
Q.CCI-E3-5D	Affx-92553843 (Exon: TraesCS5D02G226500)	C/A	CCI-Mean (Salt Stress)	277	16	19	1.89	2.1	2.41	****	0.51
Q.DB-E3-3B	Affx-92209376 (Intergenic)	C/G	DB-Mean (Control)	222	58	10	0.36	0.3	0.25	****	0.11
Q.DB-E3-1B	Affx-92766091 (Exon: TraesCS1B02G028800)	C/T	DB-Mean (Control)	173	96	35	0.30	0.3	0.52	***	0.22
Q.GLA-E3-5A.1	Affx-92187251 (Intergenic)	G/A	GLA-Mean (Control)	117	66	110	43.30	30.9	34.51	****	12.43
Q.GLA-E3-1B	Affx-92122554 (Intergenic)	G/A	GLA-Mean (Control)	89	124	71	31.09	37.2	45.01	*	13.92
Q.GLA-E3-3A	Affx-92375804 (Exon: TraesCS3A02G302000)	C/T	GLA-Mean (Control)	226	35	50	35.23	39.7	48.94	*	13.71
Q.GLA-E3-1A	Affx-92157471 (Exon: TraesCS1A02G312500)	C/T	GLA-Mean (Control)	160	147		35.08	40.5		*	5.43
Q.GLA-E3-5A.2	Affx-92660442 (Intergenic)	T/G	GLA-Mean (Salt Stress)	266	31	14	10.90	12.9	21.01	****	10.11
Q.GLA-E3-2B	Affx-92654322 (Intergenic)	G/A	GLA-Mean (Salt Stress)	247	47	3	12.39	7.7	8.94	***	4.66
Q.GLA-E3-7B.2	Affx-92751769 (Exon: TraesCS7B02G402800)	C/T	GLA-Mean (Salt Stress)	212	34	39	10.04	14.5	17.23	***	7.19
Q.GLA-E3-5D	Affx-92918236 (Exon: TraesCS5D02G513100)	C/T	GLA-Mean (Salt Stress)	81	143	71	9.29	11.1	15.12	*	5.83

#Depending on data distribution, phenotypic differences were tested using ANCOVA with population structure covariates (PC1-PC3) or with Kruskal -Wallis. Statistical significance is represented as p <0.05, *; p <0.001, **; p <0.001, ***; p < 0.0001, ****.

**Figure 7:**
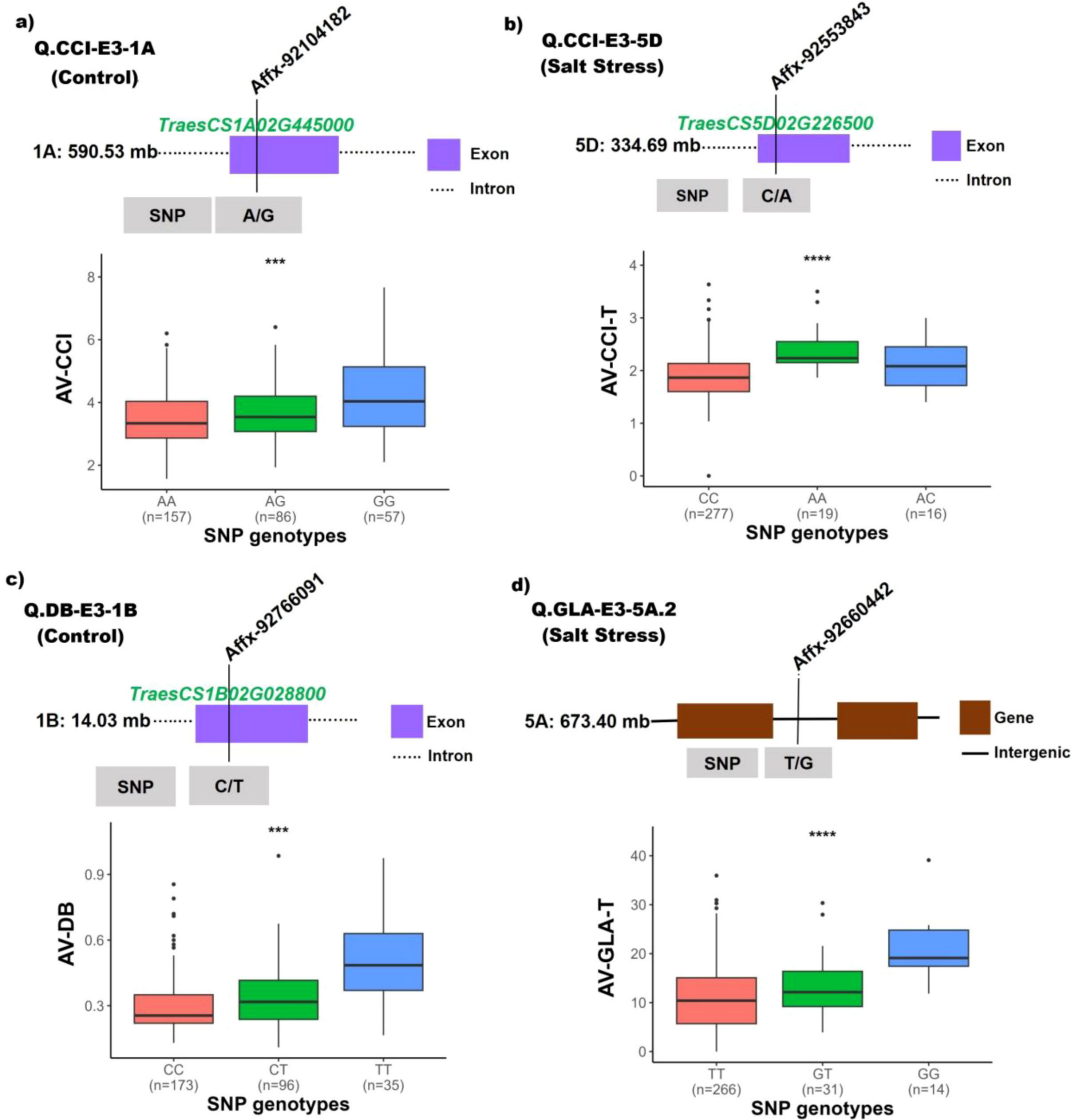
Phenotypic effects of representative GWAS-significant QTNs under control and salt stress: boxplots show trait distribution across **(A)** CCI control **(B)** CCI salt stress **(C)** DB control and **(D)** GLA salt stress. SNP positions relative to candidate genes are illustrated above each panel. Significance levels are based on Kruskal-wallis or ANCOVA (p <0.05, *; p <0.001, **; p <0.001, ***; p < 0.0001, ****).

GLA exhibited strongest genotype-dependent phenotypic effects among analysed traits, Q.GLA-E3-5A.1, Q.GLA-E3-1B and Q.GLA-E3-3A showed highly significant effects. Particularly, Q.GLA-E3-5A.1 displayed ∆Mean 12.43 indicating substantial allelic influence on canopy development. Under salt stress 4 QTNs contributed to substantial reductions in leaf area (Q.GLA-E3-5A.2, Q.GLA-E3-2B, Q.GLA-E3-7B.2, Q.GLA-E3-5D) with ∆Mean ranging from 4.66-10.11, particularly Q.GLA-E3-5A.2 affected GLA under salt stress (p <0.0001) GG genotype exhibited higher GLA relative to TT and TG genotypes indicating strong allelic contribution (**Figure 7d**). For CCI under control conditions, several SNPs exhibited significant phenotypic differentiation with ∆Mean of ~0.6-0.8. Notably, Q.CCI-E3-1A and Q.CCI-E3-4A.1 both located within annotated gene regions exhibited strong genotype effects with ∆Mean exceeding 0.7, indicating allelic influence on CCI (**Figure 7a**). Under salt stress, Q.CCI-E3-5D mapped to *TraesCS5D02G226500* displayed a highly significant effect (p < 0.0001) with ∆Mean of 0.51 highlighting a stress-specific response, plants carrying AA showed higher CCI values as compared to CC and CA genotypes suggesting enhanced chlorophyll retention under stress (**Figure 7b**). Two QTNs (Q.DB-E3-3B and Q.DB-E3-1B with ∆Mean of 0.11 and 0.22 respectively) for DB under control conditions demonstrated highly significant phenotypic differences indicating allelic contributions to biomass accumulation. Particularly, Q.DB-E3-3B located with *TraesCS1B02G028800* displayed a significant effect (p < 0.001) with ∆Mean of 0.22, the TT genotype was associated with increased biomass accumulation as compared to CC and CT genotypes (**Figure 7c**). Based on statistical significance, ∆Mean and consistency with GWAS results a subset of high-impact SNPs prioritized, many of these loci are located within or near annotated genes.

### 3.7 Identification and in silico expression analysis of candidate genes

**Figure 8:**
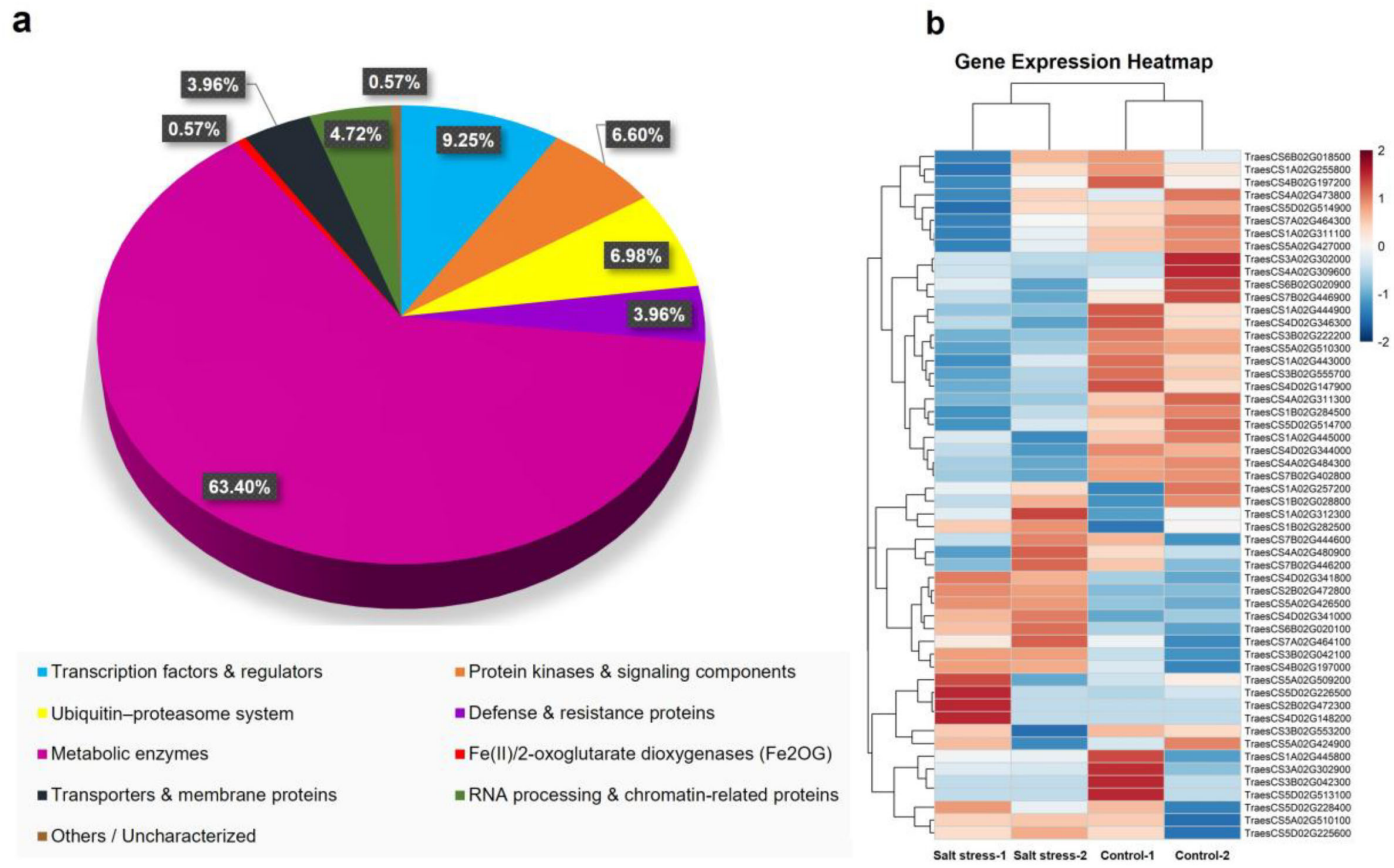
**(a)** Percentage of different gene categories within the LD **(b)** the heat map displays the expression patterns of 54 candidate genes in wheat leaf tissue under control and salt stress treatment.

Putative salt-tolerance genes were identified by aligning trait-linked SNP markers with the wheat reference genome. Genes positioned within 1.7 Mb on either side of each SNP based on LD decay estimates were considered as putative candidates for salt tolerance. A total of 530 genes were detected in the 1.7 Mb LD block that contained 24 reliable marker trait associations. Among the genes identified within the broad LD regions, the majority were classified as metabolic enzymes (63.40%) followed by transcription factors & regulators (9.25%), ubiquitin–proteasome system (6.98%), protein kinases & signaling components (6.60%), RNA processing & chromatin-related proteins (4.72%), defense & resistance proteins (3.96%), transporters & membrane proteins (3.96%), Fe (II)/2-oxoglutarate dioxygenases (Fe_2_OG) (0.57%), and uncharacterized (0.57%) (**Figure 8a**; **Supplementary Table S4**). Among these 530 genes, 67 potential expressed transcripts or putative genes were identified in association with 24 reliable QTNs or genomic regions (**Table 5**). These transcripts belonged to a wide range of functional categories, including salt-stress response proteins, calcium-dependent sensors, protein kinase domain-containing proteins, and non-specific serine/threonine protein kinases etc. The reliable genomic regions/QTNs revealed the presence of various channels and transporters, including a major facilitator superfamily (MFS) profile domain-containing protein (4A), calcium-transporting ATPase (1A), mitochondrial pyruvate carrier (4A), metal transporter (7A), ABC transporter C family member 13 (2B), mechanosensitive ion channel protein (5A), glutamate receptor (5D), mitochondrial pyruvate carrier (4A), and probable magnesium transporter (7B) (**Table 5**). Additionally, expression analysis of the candidate genes was carried out using RNA-seq data (PRJNA487922) from wheat leaves under both salt-stressed and control conditions (Amirbakhtiar et al., 2021). To assess the expression, this dataset was re-analysed using quality control, alignment, and quantification, and FPKM values of these candidate genes were used to construct the heatmap (**Figure 8b**). Twenty-eight genes showed significant upregulation under salinity stress (treatment) (**Figure 8b**). Key stress-responsive genes showed upregulated expression in leaf tissues under salinity stress, including Fe_2_OG dioxygenase domain-containing protein (Affx-92574889; *TraesCS4D02G341800*), ABC transporter C family member 13 (Affx-92654322; *TraesCS2B02G472800*), non-specific serine/threonine protein kinase (Affx-92187251; *TraesCS5A02G426500*), Homeobox domain-containing protein (Affx-92574889; *TraesCS4D02G341000*), Myb-like domain-containing protein (Affx-92760068; *TraesCS6B02G020100*), NAC023_7A.1 (Affx-92350042; *TraesCS7A02G464100*), F-box protein AT5G49610-like beta-propeller domain-containing protein (Affx-92475871; *TraesCS3B02G042100*) and Glycosyltransferases (Affx-92830496; *TraesCS4B02G197000*) in salt stress (treatment). Additionally, the spatio-temporal expression profiles of the candidate genes were examined using the Wheat Expression Browser (http://www.wheat-expression.com/). Fifty-four genes were analysed out of which 48 candidate genes showed expression in at least one tissue or developmental stage (**Supplementary Figure S2**), indicating their potential involvement in wheat growth and development.

**Table 5:** Functional annotation of candidate genes associated with reliable QTNs.

S. No.	Trait	Marker	QTN	Position (bp)	Transcript ID	Functional annotation
1	Chlorophyll content index (CCI-E3)	Affx-92104182	Q.CCI-E3-1A	1A: 592,833,080-592,846,052	*TraesCS1A02G445000*	Structural maintenance of chromosomes protein 5
				1A: 592,829,982-592,831,432	*TraesCS1A02G444900*	RING-type E3 ubiquitin transferase
				1A: 591,367,144-591,371,080	*TraesCS1A02G443000*	Calmodulin-binding domain-containing protein
				1A: 593,403,273-593,406,438	*TraesCS1A02G445800*	Rho GDP-dissociation inhibitor 1
2	Chlorophyll content index (CCI-E3)	Affx-92475871	Q.CCI-E3-3B.1	3B: 21,384,803-21,387,745	*TraesCS3B02G042100*	F-box protein AT5G49610-like beta-propeller domain-containing protein
				3B: 21,402,835-21,405,805	*TraesCS3B02G042300*	Anthranilate synthase
				3B: 22,207,886-22,210,892	*TraesCS3B02G043400*	Protein kinase domain-containing protein
3	Chlorophyll content index (CCI-E3)	Affx-92291576	Q.CCI-E3-3B.2	3B: 789,217,940-789,222,322	*TraesCS3B02G555700*	non-specific serine/threonine protein kinase
				3B: 787,850,236-787,854,279	*TraesCS3B02G553200*	Phospholipase C
4	Chlorophyll content index (CCI-E3)	Affx-92667519	Q.CCI-E3-4A.1	4A: 602,873,144-602,875,409	*TraesCS4A02G309600*	Disease resistance R13L4/SHOC-2-like LRR domain-containing protein
				4A: 601,721,888-601,725,274	*TraesCS4A02G308100*	tRNA-guanine (15) transglycosylase-like domain-containing protein
				4A: 603,458,204-603,463,050	*TraesCS4A02G311300*	Major facilitator superfamily (MFS) profile domain-containing protein
5	Chlorophyll content index (CCI-E3)	Affx-92188825	Q.CCI-E3-4A.2	4A: 734,481,412-734,484,647	*TraesCS4A02G476800*	Cytochrome P450
				4A: 733,638,666-733,640,450	*TraesCS4A02G473800*	F-box domain-containing protein
				4A: 734,481,412-734,484,647	*TraesCS4A02G475700*	R13L1/DRL21-like LRR repeat region domain-containing protein
6	Chlorophyll content index (CCI-E3)	Affx-92124522	Q.CCI-E3-4D	4D: 144,882,869-144,896,961	*TraesCS4D02G148200*	AP2/ERF domain-containing protein
				4D: 143,577,454-143,582,406	*TraesCS4D02G147900*	RNA helicase
7	Chlorophyll content index (CCI-E3)	Affx-92553843	Q.CCI-E3-5D	5D: 334,695,303-334,701,620	*TraesCS5D02G226500*	Glutamate receptor
				5D: 335,866,258-335,869,484	*TraesCS5D02G228400*	WAT1-related protein
				5D: 333,978,935-333,979,393	*TraesCS5D02G225600*	C2H2-type domain-containing protein
8	Green leaf area (GLA- E3)	Affx-92157471	Q.GLA-E3-1A	1A: 504,313,051-504,315,953	*TraesCS1A02G312500*	Uncharacterized protien
				1A: 502,804,558-502,806,739	*TraesCS1A02G311100*	Polygalacturonase
				1A: 504,270,809-504,284,581	*TraesCS1A02G312300*	Calcium-transporting ATPase
9	Green leaf area (GLA- E3)	Affx-92122554	Q.GLA-E3-1B	1B: 492,787,405-492,793,416	*TraesCS1B02G284000*	RING-type domain-containing protein
				1B: 491,250,125-491,254,720	*TraesCS1B02G282500*	RRM domain-containing protein
				1B: 493,871,055-493,873,999	*TraesCS1B02G284500*	Histone deacetylase
10	Green leaf area (GLA- E3)	Affx-92375804	Q.GLA-E3-3A	3A: 535,216,244-535,219,824	*TraesCS3A02G302000*	DUF155 domain-containing protein
				3A: 536,895,105-536,897,646	*TraesCS3A02G302900*	TCP domain-containing protein
				3A: 534,987,566-534,992,062	*TraesCS3A02G300900*	dCMP deaminase
11	Green leaf area (GLA- E3)	Affx-92170517	Q.GLA-E3-4A	4A: 737,094,089-737,097,918	*TraesCS4A02G480900*	Mitochondrial pyruvate carrier
				4A: 738,747,439-738,754,291	*TraesCS4A02G484300*	Helicase C-terminal domain-containing protein
12	Green leaf area (GLA- E3)	Affx-92830496	Q.GLA-E3-4B	4B: 424,748,291-424,750,809	*TraesCS4B02G197200*	Phosphatidylinositol transfer protein N-terminal domain-containing protein
				4B: 424,285,982-424,290,102	*TraesCS4B02G197000*	Glycosyltransferases
				4B: 424,721,434-424,722,601	*TraesCS4B02G197100*	LOB domain-containing protein
13	Green leaf area (GLA- E3)	Affx-92187251	Q.GLA-E3-5A.1	5A: 610,217,812-610,223,278	*TraesCS5A02G424900*	DNA topoisomerase
				5A: 611,564,144-611,567,697	*TraesCS5A02G426500*	Non-specific serine/threonine protein kinase
				5A: 611,817,512-611,817,955	*TraesCS5A02G427000*	VQ domain-containing protein
14	Green leaf area (GLA- E3)	Affx-92350042	Q.GLA-E3-7A	7A: 658,637,042-658,651,135	*TraesCS7A02G462400*	Uncharacterized protein
				7A: 660,550,059-660,555,686	*TraesCS7A02G464300*	Metal transporter
				7A: 660,495,082-660,499,083	*TraesCS7A02G464100*	NAC023_7A.1
15	Green leaf area (GLA- E3)	Affx-92792697	Q.GLA-E3-7B.1	7B: 708,951,404-708,955,506	*TraesCS7B02G444600*	Probable magnesium transporter
				7B: 709,608,585-709,613,542	*TraesCS7B02G446900*	Endoplasmin homolog
				7B: 709,510,490-709,514,663	*TraesCS7B02G446200*	Thaumatin-like protein
16	Green leaf area (GLA- E3)	Affx-92654322	Q.GLA-E3-2B	2B: 667,785,962-667,803,193	*TraesCS2B02G471100*	NB-ARC domain-containing protein
				2B: 669,093,154-669,094,508	*TraesCS2B02G472300*	Anthocyanin 5-aromatic acyltransferase
				2B: 669,435,939-669,444,373	*TraesCS2B02G472800*	ABC transporter C family member 13
17	Green leaf area (GLA- E3)	Affx-92660442	Q.GLA-E3-5A.2	5A: 673,402,816-673,405,436	*TraesCS5A02G509200*	60S ribosomal protein L23
				5A: 673,775,261-673,779,102	*TraesCS5A02G510100*	CCT domain-containing protein
				5A: 673,835,853-673,839,645	*TraesCS5A02G510300*	Mechanosensitive ion channel protein
18	Green leaf area (GLA- E3)	Affx-92918236	Q.GLA-E3-5D	5D: 537,161,285-537,162,607	*TraesCS5D02G513100*	Lipoxygenase
				5D: 538,420,429-538,425,328	*TraesCS5D02G514900*	TF-B3 domain-containing protein
				5D: 538,344,016-538,346,584	*TraesCS5D02G514700*	Cytochrome P450
19	Green leaf area (GLA- E3)	Affx-92751769	Q.GLA-E3-7B.2	7B: 670,278,572-670,289,261	*TraesCS7B02G402800*	MMS19 nucleotide excision repair protein
20	Dry biomass (DB-E3)	Affx-92190494	Q.DB-E3-1A	1A: 448,926,775-448,930,744	*TraesCS1A02G256600*	Zinc finger GRF-type domain-containing protein
				1A: 449,518,415-449,520,334	*TraesCS1A02G257200*	AAA+ ATPase domain-containing protein
				1A: 448,033,492-448,037,889	*TraesCS1A02G255800*	1-deoxy-D-xylulose-5-phosphate synthase
21	Dry biomass (DB-E3)	Affx-92766091	Q.DB-E3-1B	1B: 14,030,108-14,031,735	*TraesCS1B02G028800*	Pentatricopeptide repeat-containing protein
				1B: 15,097,670-15,100,117	*TraesCS1B02G030500*	DUF6598 domain-containing protein
22	Dry biomass (DB-E3)	Affx-92209376	Q.DB-E3-3B	3B: 279,960,542-279,966,047	*TraesCS3B02G222000*	Uncharacterized protein
				3B: 279,960,542-279,966,047	*TraesCS3B02G222200*	Serine aminopeptidase S33 domain-containing protein
23	Dry biomass (DB-E3)	Affx-92574889	Q.DB-E3-4D	4D: 499,502,479-499,504,778	*TraesCS4D02G344000*	Ubiquitin-like domain-containing protein
				4D: 498,572,979-498,576,397	*TraesCS4D02G341800*	Fe2OG dioxygenase domain-containing protein
				4D: 500,311,363-500,313,978	*TraesCS4D02G346300*	MADS-box domain-containing protein
				4D: 497,944,541-497,949,519	*TraesCS4D02G341000*	Homeobox domain-containing protein
24	Dry biomass (DB-E3)	Affx-92760068	Q.DB-E3-6B	6B: 11,537,907-11,540,117	*TraesCS6B02G018500*	Cytochrome P450
				6B: 12,666,214-12,673,718	*TraesCS6B02G020900*	Ubiquitin-like domain-containing protein
				6B: 12,115,521-12,119,099	*TraesCS6B02G020100*	Myb-like domain-containing protein

## 4 Discussion

The detection of genes or genomic regions linked to salt tolerance at the seedling stage can enhance the efficiency of developing high-yielding, salt-tolerant cultivars. In this context, the rich wheat germplasm collections preserved in National Gene Bank, ICAR-NBPGR, New Delhi, provides a robust genetic resource for detection genes or genomic regions through association genetic approaches. In this study, a diverse panel of 313 wheat genotypes was analysed to identify genomic regions associated with salt tolerance traits at the vegetative growth stage.

### 4.1 Variation for salt tolerance traits

The salinity stress negatively affected all measured morpho-physiological traits, which was in line with findings from previous studies (Said et al., 2022; Chaurasia et al., 2020; Khan et al., 2022, Khan et al., 2024b). Among the measured morphological traits, GLA decreased by more than 67% under treatment compared to the control. Similar reductions in leaf area under salt stress have been reported previously and are mainly attributed osmotic and ionic stresses that limit leaf expansion (Said et al., 2022; Wang et al., 2023). Salinity stress also caused a substantial decline in CCI which was decreased by more than 46% relative to the control. This observation was consistent with earlier studies reporting salinity-mediated reductions in chlorophyll content (Chaurasia et al., 2020; Said et al., 2022). The decline in chlorophyll content under salinity is largely attributed to increased production of reactive oxygen species (ROS), which causes oxidative damage and accelerates chlorophyll degradation (Taïbi et al., 2016). Furthermore, DB decreased by more than 44% under stress conditions, corroborating earlier findings that salinity severely limits biomass accumulation (Chaurasia et al., 2021; Khan et al., 2022, Khan et al., 2024a). This reduction in biomass can be explained by restricted water uptake due to osmotic stress, coupled with ionic toxicity and nutrient imbalance, ultimately constraining plant growth under saline environments (Pandit et al., 2024).

### 4.2 Linkage disequilibrium and genetic diversity

The extent of linkage disequilibrium (LD) between markers is a key factor determining mapping resolution in GWAS. In the present wheat diversity panel, the D sub-genome exhibited a more rapid LD decay compared to the A and B sub-genomes, suggesting a higher historical recombination frequency and, consequently, greater mapping resolution in this genome. Similar sub-genome-specific LD decay patterns have been reported earlier in wheat, with the D genome often showing faster LD decay than the A and B genomes (Chao et al., 2010; Hussain et al., 2022; Wu et al., 2021). Such variation in LD patterns is shaped by long-term recombination history, selection, and the genetic composition of the association panel, and therefore often differs across wheat diversity panels, containing genetically diverse and unrelated genotypes.

STRUCTURE analysis identified two distinct sub-populations among the 313 genotypes in the wheat association panel. This observation was supported by both principal component analysis (PCA) and neighbor-joining (NJ) phylogenetic analysis. However, these genetic groups did not reflect the geographical origins of the genotypes. This lack of geographic structuring is likely a consequence of extensive germplasm exchange among national and international wheat breeding programs, along with historical population movement and admixture, which together have blurred regional genetic boundaries (Pradhan et al., 2020; Chaurasia et al., 2021).

### 4.3 Association analyses

#### 4.3.1 Chlorophyll content index traits

During anthesis, chlorophyll concentration is a key determinant of photosynthetic efficiency in plants under salinity stress (Chaurasia et al., 2021). Although previous studies have mapped genes or genomic regions linked to this trait, they were conducted using a limited or less diverse set of genotypes (Chaurasia et al., 2020, Chaurasia et al., 2021; Said et al., 2022). Therefore, our objective was to identify associated genomic regions or genes using a larger and more diverse set of genotypes. In this study, we discovered three previously unreported QTNs linked to chlorophyll content, distributed across chromosomes 1A, 4D, and 5D. Among them, Q.CCI-E3-5D showed the strongest effect, explaining 10.58–11.20% of the phenotypic variation, indicating its major role in maintaining chlorophyll content under salt stress. The other two QTNs, Q.CCI-E3-1A and Q.CCI-E3-4D, explained smaller but consistent proportions of phenotypic variation, suggesting that they may act as minor yet stable contributors to chlorophyll retention. In previous studies, Chaurasia et al. (2020, Chaurasia et al., 2021) reported QTNs related to chlorophyll content on chromosomes 2AL, 4AL, 7AS, 2BS, 3BS, and 6DS, while Ilyas et al. (2020) reported QTLs on chromosome 7A. Javid et al. (2022) mapped QTLs for chlorophyll a and b on chromosomes 7B and 6A, respectively. Notably, the identification of QTNs on the D genome, particularly the major locus on chromosome 5D, is of interest because this sub-genome has been relatively less explored for stress tolerance traits. Overall, these results suggest that wheat genotypes may rely on multiple and diverse genetic mechanisms to maintain chlorophyll content under saline conditions. The use of a genetically diverse association panel therefore increases the likelihood of capturing novel alleles associated with salinity tolerance, supporting earlier observations that broader germplasm panels can reveal additional genetic variation for stress-adaptive traits (Singh et al., 2018).

#### 4.3.2 Green leaf area traits

Green leaf area plays a key role in efficient photosynthesis under salinity stress, serving as the primary surface for capturing sunlight and converting it into chemical energy (Chauhan et al., 2023). Since this trait has not been well explored under salinity stress, hence it was utilized in this study to identify novel genes and genomic regions involved in salt tolerance. In this study, 12 novel QTNs linked to green leaf area were detected on chromosomes 1A, 3A, 4A, 5A, 7A, 1B, 2B, 4B, 7B, and 5D. Among these, Q.GLA-E3-1A showed the highest contribution to phenotypic variation (7.33–14.49%), followed by Q.GLA-E3-7A, which accounted for 5.95–14.07% of the variation. These 12 novel QTNs, significantly associated with green leaf area, likely to play important roles in improving salt tolerance in certain wheat varieties.

#### 4.3.3 Dry biomass traits

Genetic regulation of total seedling DB under salinity stress is still unclear because earlier mapping efforts primarily focused on shoot and root dry weight traits individually. To date, only a limited number of studies have reported QTL mapping for this trait (Chaurasia et al., 2020; Quan et al., 2021; Javid et al., 2022; Khan et al., 2022; Javid et al., 2025). In this study, five novel QTNs associated with DB were identified on chromosomes 1A, 1B, 3B, 4D, and 6B. Among them, Q.DB-E3-1A on chromosome 1A contributed the most to phenotypic variation (3.86–18.02%), highlighting its potential significance in enhancing plant tolerance to salt stress. Previous studies reported two QTNs for shoot and root dry biomass on chromosome 3B (Javid et al., 2022), one QTN for dry root biomass on chromosome 7D (Ilyas et al., 2020), and associations with root dry biomass on chromosomes 5B and 1A (Beyer et al., 2019). These five novel QTNs may play a significant role in developing wheat varieties with enhanced salinity tolerance.

### 4.4 Candidate genes associated with salt tolerance traits

We identified various gene classes within QTNs and genomic regions associated with distinct traits related to salt tolerance. Previous studies have reported that these gene classes encode signal transduction proteins and enzymes, stress and defense related proteins, ion channels and transporters, and proteins involved in sugar accumulation, all of which collectively regulate wheat’s physiological responses to salt stress (Oyiga et al., 2018; Chaurasia et al., 2020; Chaurasia et al., 2021, Prasad et al., 2022). Notably, six potential candidate genes encoding for transporters were located within QTN GLA-E3. These included calcium-transporting ATPase (Affx-92157471; *TraesCS1A02G312300*), mitochondrial pyruvate carrier (Affx-92170517; *TraesCS4A02G480900*), metal transporter (Affx-92350042; *TraesCS7A02G464300*), ABC transporter C family member 13 (Affx-92654322; *TraesCS2B02G472800*), mechanosensitive ion channel protein (Affx-92660442; *TraesCS5A02G510300*), and probable magnesium transporter (Affx-92792697; *TraesCS7B02G444600*). These transporters are likely to play complementary roles in regulating intracellular ion homeostasis, particularly Na⁺, K⁺, Ca^2^⁺, and Mg^2^⁺, thereby supporting cellular integrity and sustaining green leaf area under salt stress. Maintenance of ion balance is a key adaptive mechanism in salt-tolerant genotypes, and the presence of multiple transporter genes within this region suggests a coordinated genetic control of this trait. In the genomic region linked to QTN CCI-E3, a major facilitator superfamily (MFS) profile domain-containing protein (Affx-92667519; *TraesCS4A02G311300*) may contribute to chlorophyll retention. MFS domain containing proteins are shown to enhance salt tolerance in plants by promoting chlorophyll retention, proline synthesis, antioxidant enzyme activity, and Na⁺/K⁺ balance (Remy et al., 2015; Li et al., 2025b). Additionally, the AP2/ERF domain-containing protein, which plays a crucial role in regulating plant responses to various abiotic stresses in plants (Yu et al., 2022), is located in the genomic region (Affx-92124522; *TraesCS4D02G148200*) associated with QTN CCI-E3 may enhance the salinity tolerance by ion homeostasis in cell.

Transcriptional regulation is reported to be key component of salt stress tolerance in crops. Eight transcription factor genes were identified across genomic regions associated with CCI-E3, GLA-E3, and DB-E3, highlighting their role in salt stress adaptation. The C2H2-type domain-containing transcription factor associated with CCI-E3 (Affx-92553843; *TraesCS5D02G225600*) may enhance salt tolerance by promoting sodium (Na⁺) excretion and reducing stomatal size. This is consistent with previous report showing that TaZNF, a member of the C2H2 zinc finger TF family improves Na⁺ excretion and reduces stomatal size under salt stress in wheat (Ma et al., 2016). The transcription factor NAC023_7A.1 (Affx-92350042; *TraesCS7A02G464100*) linked with GLA-E3, may help maintain cellular homeostasis and protect cellular components under saline stress in wheat (Traye et al., 2025). Members of the NAC transcription factor family are well known for their involvement in salinity tolerance across multiple plant species (Wang et al., 2016a). A TCP domain-containing TF (Affx-92375804; *TraesCS3A02G302900*) located within the genomic region GLA-E3 may regulate Na⁺/K⁺ balance under saline conditions, which aligns with previous findings showing that TCP TFs enhance salt tolerance in plants (Zhang et al., 2023; Wang et al., 2024b). In addition, a TF-B3 domain-containing protein (Affx-92918236; *TraesCS5D02G514900*) linked with GLA-E3 may regulate abscisic acid (ABA) signaling and oxidative stress responses, as shown in eggplant under salt stress (Ding et al., 2023). Similarly, LOB domain-containing protein (Affx-92830496; *TraesCS4B02G197100*) associated with GLA-E3, suggests a potential role in regulating ROS levels in leaves, which may enhance salinity tolerance in multiple plant species (Wang et al., 2010, Wang et al., 2024a; Guan et al., 2023).

Beyond transporters and transcription factors, several additional candidate genes with known roles in stress adaptation were detected. These included pentatricopeptide repeat proteins involved in water balance regulation, homeobox domain-containing proteins linked to stress resilience, and multiple stress-responsive enzymes such as RING-type E3 ubiquitin ligases, calmodulin-binding proteins, F-box proteins, and protein kinases potentially associated with the SOS signaling pathway (Deng et al., 2013; Jia et al., 2015; Jiang et al., 2015; Li et al., 2022, Li et al., 2025a; Shen et al., 2023).

The haplo-pheno analysis revealed several candidate genes, including the structural maintenance of chromosomes protein 5 (*TraesCS1A02G445000*) linked with CCI-E3, involved in regulating both abiotic and biotic stress responses in plants (Diaz and Pecinka, 2018). The glutamate receptor gene (*TraesCS5D02G226500*) also associated with CCI-E3 and plays a key role in Ca^2^⁺ signaling and ion influx, contributing to the regulation of reactive oxygen species (ROS) generated during salt stress (Lindberg and Premkumar, 2023) and this gene was also upregulated under salt stress in *in-silico* expression analysis. The pentatricopeptide repeat-containing protein (*TraesCS1B02G028800*) linked with DB-E3, enhances salt tolerance by increasing abscisic acid (ABA) sensitivity and improving stress management in plants (Lu et al., 2022; Jiang et al., 2015). Additional key candidate genes associated with stress tolerance were also identified, including those encoding a disease resistance R13L4/SHOC-2–like LRR domain–containing protein, a DUF155 domain–containing protein, and lipoxygenase (Yang et al., 2025; Waseem et al., 2021; Menga and Trono, 2020).

Collectively, the identification of these functionally diverse yet interconnected candidate genes highlight the complex genetic architecture underlying salt tolerance in wheat. These genes represent strong targets for functional validation and could be effectively exploited in marker-assisted selection and genomics-assisted breeding programs aimed at developing salt-tolerant wheat cultivars.

## 5 Conclusion

This study revealed key molecular and genetic factors associated with traits that confer salt tolerance in wheat under prolonged salinity stress. Twenty-four significant QTNs were identified using two or more models, four of which had a major impact on their respective salt tolerance traits. Several potential candidate genes were also discovered within the associated genomic regions. Four reliably identified QTNs were significantly associated with selected traits, including CCI (Q.CCI-E3-1A, Q.CCI-E3-5D), DB (Q.DB-E3-1B), and GLA (Q.GLA-E3-5A.2). The newly identified genes and genomic regions from this study may serve as valuable targets for developing salt-tolerant wheat varieties.

## Data Availability

The original contributions presented in the study are included in the article/**Supplementary Material**. Further inquiries can be directed to the corresponding author.

## Supplementary material

Supplementary Figure S1 Experimental setup showing 313 wheat genotypes grown under hydroponic conditions in Hoagland nutrient solution under control and salinity treatment (15 dS m^-1^) using NaCl, CaCl_2_·2H_2_O, and Na_2_SO_4_.

Supplementary Figure S2 A heat map was generated to illustrate the spatial and temporal expression patterns of the candidate genes using the Wheat Expression Browser. A total of 54 candidate genes were analysed, of which 48 showed expression. Gene expression was examined across multiple tissues and developmental stages, including root, stem, leaf, spike, and grain.

Supplementary Table S1 Details of the 313 wheat accessions used in this study.

Supplementary Table S2 Twenty-five salt tolerance QTNs identified in control condition using two or more than two multi-locus GWAS models.

Supplementary Table S3 Nineteen salt tolerance QTNs identified in treatment condition using two or more than two multi-locus GWAS models.

Supplementary Table S4 Gene categories and number of genes detected within the LD.
