# Supplementary material for: Genome-wide association studies and candidate gene identification under salinity stress in bread wheat (Triticum aestivum L.)
Source: Front Plant Sci. 2026 Apr 22;17:1817999. doi: 10.3389/fpls.2026.1817999 (PMC13148073; doi:10.3389/fpls.2026.1817999)
Supplement: Supplementary Figure S1 — Experimental setup showing 313 wheat genotypes grown under hydroponic conditions in Hoagland nutrient solution under control and salinity treatment (15 dS m-1) using NaCl, CaCl2·2H2O, and Na2SO4. [file Image1.pdf]

Supplementary Figures

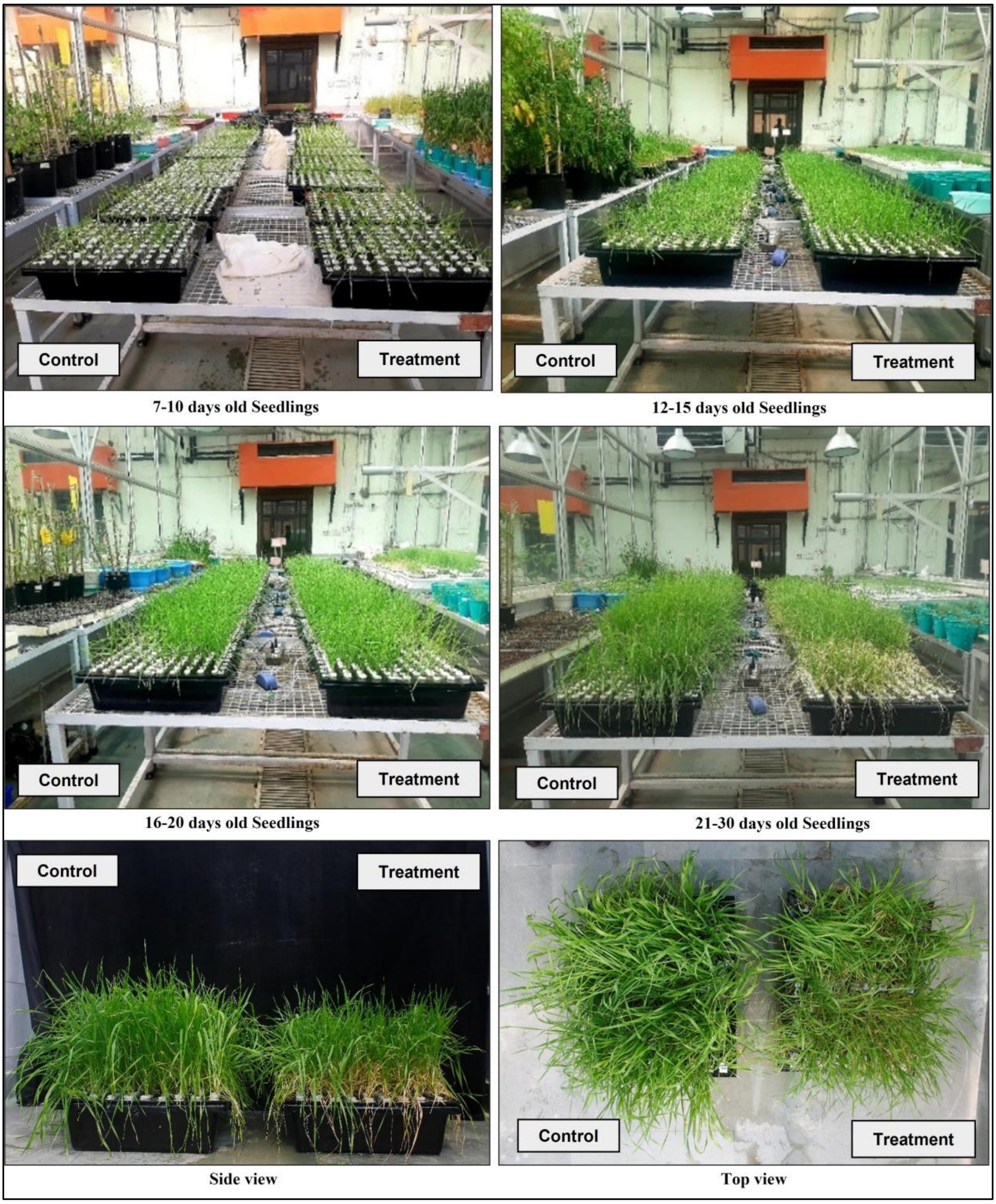

Supplementary Figure S1: Experimental setup showing 313 wheat genotypes grown under hydroponic conditions in Hoagland nutrient solution under control and salinity treatment (15 dS m^{-1}) using NaCl, $CaCl_2 \cdot 2H_2O$, and Na_2SO_4.

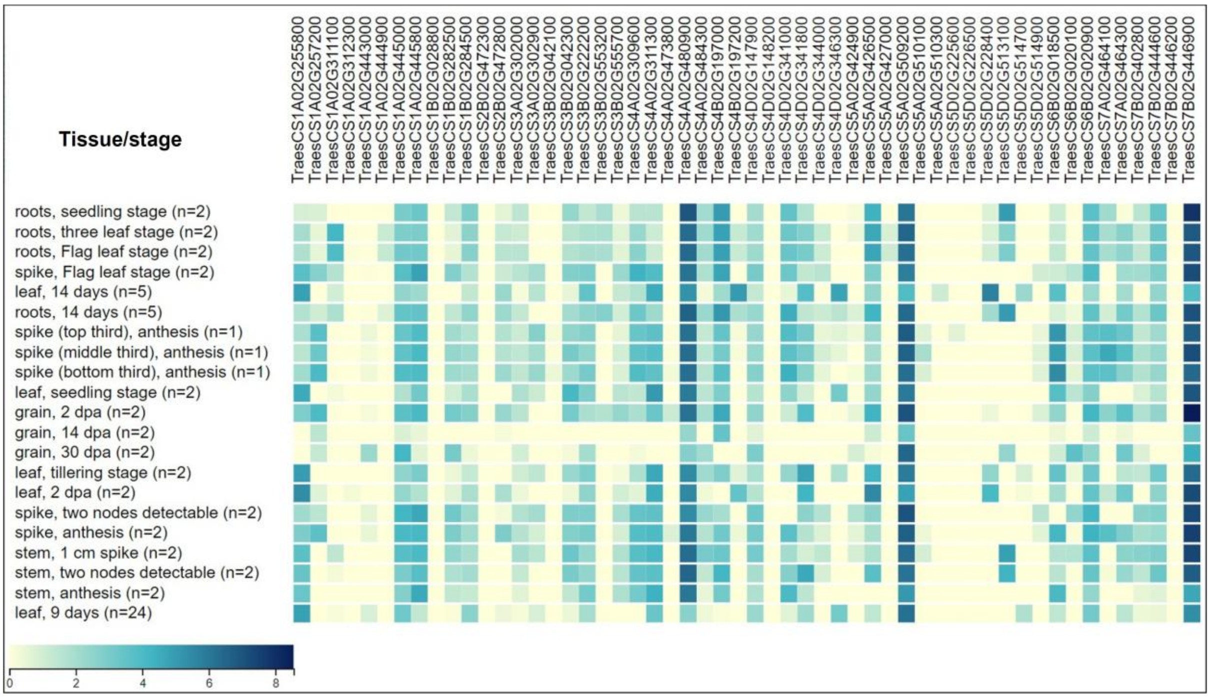

Supplementary Figure S2. A heat map was generated to illustrate the spatial and temporal expression patterns of the candidate genes using the Wheat Expression Browser. Gene expression was examined across multiple tissues and developmental stages, including root, stem, leaf, spike, and grain.
