# Supplementary material for: Genome-wide association studies and candidate gene identification under salinity stress in bread wheat (Triticum aestivum L.)
Source: Front Plant Sci. 2026 Apr 22;17:1817999. doi: 10.3389/fpls.2026.1817999 (PMC13148073; doi:10.3389/fpls.2026.1817999)
Supplement: Supplementary Table S1 — Details of the 313 wheat accessions used in this study. [file Table1.docx]

**Supplementary Tables**

**Table S1:** Details of the 313 wheat accessions used in this study.

| **S. No.** | **Accession** | **Collector No/Other ID/Cultivar name/Pedigree** | **Bio Status** | **Collection source/ developer institution /country** |
| --- | --- | --- | --- | --- |
| 1 | EC273814 | NA | NA | MEXICO |
| 2 | EC38113 | ACC - 861 | NA | USA |
| 3 | EC463384 | OASIS F 86 | NA | MEXICO |
| 4 | EC463388 | S-17 | NA | MEXICO |
| 5 | EC479350 | WQL3H-F254H | NA | USA |
| 6 | EC483002 | NA | NA | MEXICO |
| 7 | EC534451 | PAU 308 | NA | USA |
| 8 | EC534495 | PAU 695 | NA | USA |
| 9 | EC552086 | FRET2/KURUKU/FRET2 | NA | MEXICO |
| 10 | EC556440 | MN75136/PGO | NA | AUSTRALIA |
| 11 | EC556473 | NA | NA | AUSTRALIA |
| 12 | EC556482 | NA | NA | AUSTRALIA |
| 13 | EC609338 | 07A 37 | NA | USA |
| 14 | EC637483 | NA | VARIETY | Mexico |
| 15 | EC6903 | NA | NA | USA |
| 16 | EC697721 | BL 3555 | NA | NEPAL |
| 17 | IC0128198 | HUW 206 | Indian released variety | Varanasi, Uttar Pradesh, India |
| 18 | IC0128242 | PBW- 65 | Indian released variety | Ludhiana, Punjab, India |
| 19 | IC0252612 | HD-2402 HD 2267 x HD 2236 (HD 2177 x HD 2120/ HD 2160) | Indian released variety | India |
| 20 | IC0290196 | NA | Indian released variety | Karnal, Haryana, India |
| 21 | IC0296308 | NA | Indian released variety | Indore, Madhya Pradesh, India |
| 22 | IC0303072 | GW-322 | Indian released variety | India |
| 23 | IC0401942 | B1153/CB 85 = [(CHD 2402/CPAN 1830)]/VEE `S` | Indian released variety | Haryana, India |
| 24 | IC0443723 | CPAN-6018/2* RAJ1555 | Indian released variety | Dharwad, Karnataka, India |
| 25 | IC0443726 | HD-2610 | Indian released variety | New Delhi, India |
| 26 | IC0443766 | HD 2402/VL 639 | Indian released variety | Dungarpur, Rajasthan, India |
| 27 | IC0534533 | PI-183523 | NA | India |
| 28 | IC0534590 | PI-266868 | NA | India |
| 29 | IC0534597 | PI-266875 | NA | India |
| 30 | IC0534715 | PI-322127 | NA | India |
| 31 | IC0574250 | NA | Indian released variety | Madhya Pradesh, India |
| 32 | IC0595232 | PBW 343 x HW 3083 | Indian released variety | Tamil Nadu, India |
| 33 | IC0598726 | THELIN//2*ATTILA*2/PASTOR | Indian released variety | Faizabad, Uttar Pradesh, India |
| 34 | IC0611310 | (TOB/ERA//TOB/CNO67/3/PLO/4/VEE#5/5/ KAUZ/6/FRET2) / DWR-162 | Indian released variety | Dharwad, Karnataka, India |
| 35 | IC079082 | BDJ-I-1049 | Landraces local collection | Bilaspur, Himachal Pradesh, India |
| 36 | IC082265 | BDJ-86-312 | Landraces local collection | Shimla, Himachal Pradesh, India |
| 37 | IC082308 | BDJ-3258 | Landraces local collection | Solan, Himachal Pradesh, India |
| 38 | IC107399 | RKT-89/177 | Landraces local collection | Hamirpur, Himachal Pradesh, India |
| 39 | IC111703 | NA | NA | India |
| 40 | IC111738 | HI-778 | NA | India |
| 41 | IC111839 | HP-274 | NA | India |
| 42 | IC111867 | NA | NA | India |
| 43 | IC111914 | AC-214 | NA | India |
| 44 | IC118721 | SN/VKP/506 | NA | Kargil, Jammu and Kashmir, India |
| 45 | IC128177 | HD 2380 | NA | Delhi, India |
| 46 | IC128386 | NA | Landraces local collection | New Delhi, India |
| 47 | IC128640 | KA90168 | NA | Kanpur, Uttar Pradesh, India |
| 48 | IC128650 | KA90181 | NA | Kanpur, Uttar Pradesh, India |
| 49 | IC128654 | KA90186 | NA | Kanpur, Uttar Pradesh, India |
| 50 | IC138337 | NA | Landraces local collection | Indore, Madhya Pradesh, India |
| 51 | IC138379 | GL-20 | NA | Bagalkot, Karnataka, India |
| 52 | IC138384 | GL-27 | NA | Bagalkot, Karnataka, India |
| 53 | IC138426 | KA-9018 | NA | Kanpur, Uttar Pradesh, India |
| 54 | IC138553 | HP-Local-7 | Landraces local collection | Kullu, Himachal Pradesh, India |
| 55 | IC138554 | HP-Local-8 | NA | Kullu, Himachal Pradesh, India |
| 56 | IC144903 | NA | NA | India |
| 57 | IC145954 | V-878 | NA | Uttar Pradesh, India |
| 58 | IC145972 | NA | Landraces local collection | Uttar Pradesh, India |
| 59 | IC145983 | NA | Landraces local collection | Uttar Pradesh, India |
| 60 | IC240802 | 33682-L-1Y-1Y-1M-3Y-100B-503Y-500B-OY | NA | Faizabad, Uttar Pradesh, India |
| 61 | IC252349 | APAN-1905 | NA | Uttar Pradesh, India |
| 62 | IC252379 | BL-1902 | NA | Uttar Pradesh, India |
| 63 | IC252431 | BW/SH-30 | NA | West Bengal, India |
| 64 | IC252587 | HB-316 | NA | India |
| 65 | IC252644 | HDR-151 | NA | India |
| 66 | IC252660 | HP-1529 | NA | India |
| 67 | IC252714 | HUW-326 | NA | India |
| 68 | IC252784 | K-8804 | NA | India |
| 69 | IC252785 | K-8905 | NA | India |
| 70 | IC252792 | K-9325 | NA | India |
| 71 | IC252824 | MUSK-6 | NA | India |
| 72 | IC252844 | NEPAL-22 | NA | India |
| 73 | IC252853 | NI-8858 | NA | India |
| 74 | IC252876 | PBN-215 | NA | India |
| 75 | IC252887 | PBW-358 | NA | India |
| 76 | IC252918 | RAJ-3461 | NA | India |
| 77 | IC252927 | RW-346 JANAK/SA42 | NA | India |
| 78 | IC252928 | RW-482 | NA | India |
| 79 | IC252948 | UP-2358 | NA | India |
| 80 | IC252974 | VL-791 | NA | India |
| 81 | IC260894 | KCM/BDP-50 | Landraces local collection | Rudraprayag, Uttarakhand, India |
| 82 | IC260895 | KCM/BDP-51 | Landraces local collection | Rudraprayag, Uttarakhand, India |
| 83 | IC260970 | KCM/BDP-126 | Landraces local collection | Uttarkashi, Uttarakhand, India |
| 84 | IC261932 | SRB-79 | Landraces local collection | Patan, Gujarat, India |
| 85 | IC262732 | BDS/SG2982 | Landraces local collection | Mandi, Himachal, Pradesh, India |
| 86 | IC262792 | BDS/SG2997 | Landraces local collection | Bilaspur, Himachal Pradesh, India |
| 87 | IC262796 | BDS/SG2999 | Landraces local collection | Bilaspur, Himachal Pradesh, India |
| 88 | IC262899 | BDS/SG3003 | Landraces local collection | Bilaspur, Himachal Pradesh, India |
| 89 | IC266140 | VRK-198 | Landraces local collection | Almora, Uttarakhand, India |
| 90 | IC26729 | NA | NA | Lahaul & Spiti, Himachal Pradesh, India |
| 91 | IC278681 | SKY/SNS-359 | Landraces local collection | Sirmaur, Himachal Pradesh, India |
| 92 | IC279875 | RKS/UKP-262 | Landraces local collection | Chamba, Himachal Pradesh, India |
| 93 | IC281549 | VR-CW-1639 | Landraces local collection | Chamoli, Uttarakhand, India |
| 94 | IC281550 | VR-CW-1640 | Landraces local collection | Chamoli, Uttarakhand, India |
| 95 | IC281555 | VR-CW-1645 | Landraces local collection | Rudraprayag, Uttarakhand, India |
| 96 | IC281560 | VR-CW-1650 | Landraces local collection | Uttarkashi, Uttarakhand, India |
| 97 | IC281569 | VR-CW-1659 | Landraces local collection | Tehri Garhwal, Uttarakhand, India |
| 98 | IC282860 | VRB-CW-1282 | Landraces local collection | Almora, Uttarakhand, India |
| 99 | IC282871 | VRB-CW-1293 | Landraces local collection | Dehradun, Uttarakhand, India |
| 100 | IC28526 | NA | NA | Jamnagar, Gujarat, India |
| 101 | IC28628 | NA | NA | Sabarkantha, Gujarat, India |
| 102 | IC28634 | NA | NA | Sabarkantha, Gujarat, India |
| 103 | IC28749 | NA | NA | Mehsana, Gujarat, India |
| 104 | IC29002 | Raj-234 | NA | Bharatpur, Rajasthan, India |
| 105 | IC29020 | Raj-253 | NA | Bikaner, Rajasthan, India |
| 106 | IC290213 | K-8806 | NA | Shimla, Himachal Pradesh, India |
| 107 | IC296529 | K134 (60)/ VEE /BOW/ PVN /PBW 343 | NA | India |
| 108 | IC296538 | HM 00524 A | NA | India |
| 109 | IC296743 | NA | NA | India |
| 110 | IC310124 | GSS-114 | Landraces local collection | Himachal Pradesh, India |
| 111 | IC310127 | GSS-119 | Landraces local collection | Kullu, Himachal Pradesh, India |
| 112 | IC316091 | VRS-CW-1880 | Landraces local collection | Udham Singh Nagar, Uttarakhand, India |
| 113 | IC316092 | VRS-CW-1881 | Landraces local collection | Udham Singh Nagar, Uttarakhand, India |
| 114 | IC316096 | VRS-CW-1885 | Landraces local collection | Udham Singh Nagar, Uttarakhand, India |
| 115 | IC316098 | VRS-CW-1887 | Landraces local collection | Udham Singh Nagar, Uttarakhand, India |
| 116 | IC316104 | VRS-CW-1893 | Landraces local collection | Udham Singh Nagar, Uttarakhand, India |
| 117 | IC317610 | DARL/BK/561 | Landraces local collection | Pakur, Jharkhand, India |
| 118 | IC32039 | NA | NA | Satna, Madhya Pradesh, India |
| 119 | IC321153 | SKY/SNS-651 | Landraces local collection | Sirmaur, Himachal Pradesh, India |
| 120 | IC321154 | SKY/SNS-674 | Landraces local collection | Sirmaur, Himachal Pradesh, India |
| 121 | IC321847 | SK-2 | Landraces local collection | Pakur, Jharkhand, India |
| 122 | IC321851 | SK-6 | NA | Pakur, Jharkhand, India |
| 123 | IC321884 | SK-39 | NA | Pakur, Jharkhand, India |
| 124 | IC321910 | SK-65 | NA | Pakur, Jharkhand, India |
| 125 | IC321916 | SK-71 | NA | Pakur, Jharkhand, India |
| 126 | IC321919 | SK-74 | NA | Pakur, Jharkhand, India |
| 127 | IC321956 | SK-111 | NA | Madhya Pradesh, India |
| 128 | IC32788 | WIC-402 | NA |  |
| 129 | IC328326 | RK-20 | Landraces local collection | Mandi, Himachal Pradesh, India |
| 130 | IC328434 | SAW/GML-3 | Landraces local collection | Kathua, Jammu and Kashmir, India |
| 131 | IC329599 | LC-48 | Landraces local collection | Lahaul & Spiti, Himachal Pradesh, India |
| 132 | IC335679 | EGPSN (3)-64 | NA | India |
| 133 | IC335683 | EGPSN (3)-71 | NA | India |
| 134 | IC335684 | EGPSN (3)-72 | NA | India |
| 135 | IC335760 | SAWSN (17)-59 | NA | India |
| 136 | IC335977 | UDND/01-145 | Landraces local collection | Belgaum, Karnataka, India |
| 137 | IC336648 | NA | NA | Kullu, Himachal Pradesh, India |
| 138 | IC341374 | RKS/RRA-114 | Landraces local collection | Tehri Garhwal, Uttarakhand, India |
| 139 | IC345589 | (E2001-BHW-29) KJ-125 | NA | Dehradun, Uttarakhand, India |
| 140 | IC345620 | (E2001-BHW-29) KJ-156 | Landraces local collection | Dehradun, Uttarakhand, India |
| 141 | IC346055 | JCR-763 | Landraces local collection | Kangra, Himachal Pradesh, India |
| 142 | IC355866 | VRH-CW-2580 | Landraces local collection | Almora, Uttarakhand, India |
| 143 | IC356111 | BBRN-2591 | Landraces local collection | Pithoragarh, Uttarakhand, India |
| 144 | IC361690 | AK/YK-92 | Landraces local collection | Chamba, Himachal Pradesh, India |
| 145 | IC372745 | KRR/AK-109 | Landraces local collection | Kullu, Himachal Pradesh, India |
| 146 | IC375940 | HPKD/03-74 | Landraces local collection | Dharwad, Karnataka, India |
| 147 | IC376242 | HPKD/03-112 | Landraces local collection | Bijapur, Karnataka, India |
| 148 | IC382720 | AKS/TRS-1216 | Landraces local collection | Chamba, Himachal Pradesh, India |
| 149 | IC384541 | VK-SK00-110-B | NA | Dindori, Madhya Pradesh, India |
| 150 | IC384555 | VK-SK00-312 | Landraces local collection | Sagar, Madhya Pradesh, India |
| 151 | IC393124 | VR/CW-2812 | Landraces local collection | Chamoli, Uttarakhand, India |
| 152 | IC393129 | VR/CW-2817 | Landraces local collection | Chamoli, Uttarakhand, India |
| 153 | IC393880 | HD-1925/HD832//23584 | NA | Hisar, Haryana, India |
| 154 | IC394806 | SMBR-682 | NA | Assam, India |
| 155 | IC397214 | DD-4 | Landraces local collection | Indore, Madhya Pradesh, India |
| 156 | IC397815 | YS/RC-1 | NA | Bilaspur, Himachal Pradesh, India |
| 157 | IC397820 | YS/RC-6 | Landraces local collection | Bilaspur, Himachal Pradesh, India |
| 158 | IC397868 | YS/RC-54 | NA | Himachal Pradesh, India |
| 159 | IC397989 | YS/RC-178 | Landraces local collection | Bilaspur, Himachal Pradesh, India |
| 160 | IC398277 | VAH-CW-3167 | Landraces local collection | Bageshwar, Uttarakhand, India |
| 161 | IC398279 | VAH-CW-3169 | Landraces local collection | Bageshwar, Uttarakhand, India |
| 162 | IC398298 | VAH-CW-3188 | Landraces local collection | Bageshwar, Uttarakhand, India |
| 163 | IC398310 | VAH-CW-3200 | Landraces local collection | Bageshwar, Uttarakhand, India |
| 164 | IC401927 | GW-18 (J-18) S. 331 x NP 890 | NA | Haryana, India |
| 165 | IC402016 | D-84 | Landraces local collection | Haryana, India |
| 166 | IC402030 | SK-200A | Landraces local collection | Haryana, India |
| 167 | IC402033 | DWR-246 | Landraces local collection | Haryana, India |
| 168 | IC406690 | KCM-671 | Landraces local collection | Champawat, Uttarakhand, India |
| 169 | IC415868 | PAU – 190 | NA | Ludhiana, Punjab, India |
| 170 | IC415876 | PAU – 216 | NA | Ludhiana, Punjab, India |
| 171 | IC415877 | PAU – 217 | NA | Ludhiana, Punjab, India |
| 172 | IC415939 | PAU – 355 | NA | Ludhiana, Punjab, India |
| 173 | IC415955 | PAU – 375 | NA | Ludhiana, Punjab, India |
| 174 | IC415967 | PAU – 387 | NA | Ludhiana, Punjab, India |
| 175 | IC416013 | PAU – 433 | NA | Ludhiana, Punjab, India |
| 176 | IC416026 | PAU – 446 | NA | Ludhiana, Punjab, India |
| 177 | IC416046 | PAU – 466 | NA | Ludhiana, Punjab, India |
| 178 | IC416053 | PAU – 473 | NA | Ludhiana, Punjab, India |
| 179 | IC416079 | PAU – 499 | NA | Ludhiana, Punjab, India |
| 180 | IC416146 | PAU – 566 | NA | Ludhiana, Punjab, India |
| 181 | IC416168 | PAU – 588 | NA | Ludhiana, Punjab, India |
| 182 | IC416408 | SCA(A) / PAU - 983 | NA | Ludhiana, Punjab, India |
| 183 | IC418384 | KJ-540 | Landraces local collection | Chamoli, Uttarakhand, India |
| 184 | IC418402 | KJ-558 | Landraces local collection | Chamoli, Uttarakhand, India |
| 185 | IC418410 | KJ-566 | Landraces local collection | Chamoli, Uttarakhand, India |
| 186 | IC421880 | SKY/AK - 1339 | Landraces local collection | Solan, Himachal Pradesh, India |
| 187 | IC421928 | SKY/AK - 1387 | Landraces local collection | Solan, Himachal Pradesh, India |
| 188 | IC426359 | SN-072 | NA | Adilabad, Telangana, India |
| 189 | IC427213 | JCR-1004 | Landraces local collection | Himachal Pradesh, India |
| 190 | IC430349 | KCM-734 | Landraces local collection | Pauri Garhwal, Uttarakhand, India |
| 191 | IC430359 | KCM-744 | Landraces local collection | Pauri Garhwal, Uttarakhand, India |
| 192 | IC436069 | HW 2042 | NA | Tamil Nadu, India |
| 193 | IC443728 | PTO-1/CNO 79/PRL/GAA/3HD 1951 | NA | New Delhi, India |
| 194 | IC443737 | Unnath Kalyan Sona * 2//CPAN 3057 | NA | India |
| 195 | IC443738 | C-306 *7//TR 380-14#7/3 AG 14 | NA | India |
| 196 | IC443748 | K-573-10 | NA | Kanpur, Uttar Pradesh, India |
| 197 | IC443750 | K-9266 (DEWA) | NA | Kanpur, Uttar Pradesh, India |
| 198 | IC443767 | RS-31-1 | NA | Dungarpur, Rajasthan, India |
| 199 | IC445297 | ET-94301 | NA | Karnal, Haryana, India |
| 200 | IC445331 | 11th Sawyt-6 | NA | Karnal, Haryana, India |
| 201 | IC445332 | 11th Sawyt-10 | NA | Karnal, Haryana, India |
| 202 | IC445400 | ET-96195 | NA | Karnal, Haryana, India |
| 203 | IC447522 | BIJ-40 | Landraces local collection | Sirmaur, Himachal Pradesh, India |
| 204 | IC449213 | UC/TRS-31 | Landraces local collection | Chamba, Himachal Pradesh, India |
| 205 | IC469485 | VHC(BD)-67 | NA | Tehri Garhwal, Uttarakhand, India |
| 206 | IC47797 | NA | NA | Baramula, Jammu and Kashmir, India |
| 207 | IC524220 | KCM-781 | Landraces local collection | Pauri Garhwal, Uttarakhand, India |
| 208 | IC524291 | HW 2003 | NA | Wellington, Tamil Nadu, India |
| 209 | IC527448 | HI 1182/CPAN 1990 | NA | Indore, Madhya Pradesh, India |
| 210 | IC528993 | VWFW-222 | NA | Almora, Uttarakhand, India |
| 211 | IC529285 | VWFW-2180 | NA | Almora, Uttarakhand, India |
| 212 | IC529289 | VWFW-2184 | NA | Almora, Uttarakhand, India |
| 213 | IC529309 | VWFW-2204 | NA | Almora, Uttarakhand, India |
| 214 | IC529517 | VWFW-2256 | NA | Almora, Uttarakhand, India |
| 215 | IC530051 | VWFW- 2366 | NA | Almora, Uttarakhand, India |
| 216 | IC531219 | W 1030 | NA | India |
| 217 | IC531233 | ML 311 | NA | India |
| 218 | IC531275 | ML 193874 | NA | India |
| 219 | IC531363 | W 2556 | NA | India |
| 220 | IC531505 | W 6202 | NA | India |
| 221 | IC531792 | NA | Landraces local collection | India |
| 222 | IC531861 | GW-9715 | NA | India |
| 223 | IC531927 | HPW-57 | NA | India |
| 224 | IC531950 | HUW-299 | NA | India |
| 225 | IC531961 | HUW-441 | NA | India |
| 226 | IC532000 | K-9223 | NA | India |
| 227 | IC532001 | K-9228 | NA | India |
| 228 | IC532045 | NI-9463 | NA | India |
| 229 | IC532099 | P-2583 | Landraces local collection | India |
| 230 | IC532149 | P-2855 | Landraces local collection | India |
| 231 | IC532414 | P-4723 | Landraces local collection | India |
| 232 | IC532489 | P-5102 | Landraces local collection | India |
| 233 | IC532495 | P-5125 | Landraces local collection | India |
| 234 | IC532562 | PAKISTAN-81 | NA | India |
| 235 | IC532662 | UP-2296 | NA | India |
| 236 | IC532672 | UP-2377 | NA | India |
| 237 | IC532674 | UP-2388 | NA | India |
| 238 | IC532807 | WIC-411 | Landraces local collection | India |
| 239 | IC532871 | WIC-442(MICRO) | NA | India |
| 240 | IC532880 | WIC-447 | Landraces local collection | India |
| 241 | IC532928 | WIC-54 | Landraces local collection | India |
| 242 | IC533590 | PI-342966 | NA | India |
| 243 | IC533608 | PI-430035 | NA | India |
| 244 | IC533725 | 8-HRWYT-23 | NA | India |
| 245 | IC533733 | 8-SAWYT-18 | NA | India |
| 246 | IC533754 | B-26 C-4 | NA | India |
| 247 | IC533844 | CITR-5426 | NA | India |
| 248 | IC534300 | PI-176206 | NA | India |
| 249 | IC534926 | PI-348980 | NA | India |
| 250 | IC534989 | PI-430034 | NA | India |
| 251 | IC535402 | NC-59561 | NA | India |
| 252 | IC535509 | NC-60674 | NA | India |
| 253 | IC535601 | WL 410 | NA | India |
| 254 | IC535693 | PBW91 | NA | India |
| 255 | IC535710 | PBW131 | NA | India |
| 256 | IC536111 | WL 3892 | NA | India |
| 257 | IC536225 | ML 5119 | NA | India |
| 258 | IC536311 | NC-55636 | NA | India |
| 259 | IC536321 | NC-59610 | NA | India |
| 260 | IC536474 | HD-4594 | NA | India |
| 261 | IC536488 | HD-2379 | NA | India |
| 262 | IC538625 | KP/RC/05-142 | Landraces local collection | Solan, Himachal Pradesh, India |
| 263 | IC538692 | KP/RC/05-209 | Landraces local collection | Una, Himachal Pradesh, India |
| 264 | IC539184 | EIGN-I-(04-05)/99 | NA | Karnal, Haryana, India |
| 265 | IC539208 | EIGN-I-(04-05)/127 | NA | Karnal, Haryana, India |
| 266 | IC539334 | NA | NA | Karnal, Haryana, India |
| 267 | IC539415 | BCN-179 | NA | Karnal, Haryana, India |
| 268 | IC542012 | 22-SAWSN-130 | NA | Delhi, India |
| 269 | IC542045 | MEIIQ-03-6 | NA | Delhi, India |
| 270 | IC542050 | MIYCSN-19 | NA | Delhi, India |
| 271 | IC542051 | MIYCSN-22 | NA | Delhi, India |
| 272 | IC542052 | MIYCSN-26 | NA | Delhi, India |
| 273 | IC542053 | MIYCSN-32 | NA | Delhi, India |
| 274 | IC542063 | MFSYCINT-166 | NA | Delhi, India |
| 275 | IC542068 | 15-HRWSN-119 | NA | Delhi, India |
| 276 | IC543373 | IDTN70 (ET90550) | NA | India |
| 277 | IC549471 | SK-16-45-1-5 | NA | Karnal, Haryana, India |
| 278 | IC549472 | SK-46-45-1-1 | NA | Karnal, Haryana, India |
| 279 | IC549527 | WR-738 | NA | Karnal, Haryana, India |
| 280 | IC551375 | 8A-RED | NA | Karnal, Haryana, India |
| 281 | IC551394 | AC-82 | NA | Karnal, Haryana, India |
| 282 | IC554661 | WH 1021 | NA | Hisar, Haryana, India |
| 283 | IC560681 | JG 3211 | NA | Jabalpur, Madhya Pradesh, India |
| 284 | IC564138 | KCM/PSM/RK-1099 | Landraces local collection | Pauri Garhwal, Uttarakhand, India |
| 285 | IC564166 | KCM/PSM/RK-1127 | Landraces local collection | Pauri Garhwal, Uttarakhand, India |
| 286 | IC573155 | MPO-26 | Landraces local collection | Nainital, Uttarakhand, India |
| 287 | IC573902 | NA | NA | Srinagar, Jammu and Kashmir, India |
| 288 | IC582710 | HPW - 255 | NA | Kangra, Himachal Pradesh, India |
| 289 | IC582716 | HPW - 282 | Landraces local collection | Kangra, Himachal Pradesh, India |
| 290 | IC582907 | AKAW-3717 HW-2035/NI-5439 | NA | Akola, Maharashtra, India |
| 291 | IC585637 | MMBO-3029 | NA | Begeshwar, Uttarakhand, India |
| 292 | IC585652 | MMBO-3046 | Landraces local collection | Begeshwar, Uttarakhand, India |
| 293 | IC585933 | CMH-771-917-BOW/HW 2008 | NA | Maharashtra, India |
| 294 | IC589278 | TMOB-3109 | Landraces local collection | Uttarkashi, Uttarakhand, India |
| 295 | IC589295 | TMOB-3128 | Landraces local collection | Uttarkashi, Uttarakhand, India |
| 296 | IC589296 | TMOB-3129 | Landraces local collection | Uttarkashi, Uttarakhand, India |
| 297 | IC589301 | TMOB-3134 | Landraces local collection | Uttarakashi, Uttarakhand, India |
| 298 | IC595396 | TR-83 | Landraces local collection | Almora, Uttarakhand, India |
| 299 | IC598261 | DPS/OPD-46 | Landraces local collection | West Bengal, India |
| 300 | IC598262 | DPS/OPD-48 | Landraces local collection | West Bengal, India |
| 301 | IC598266 | DPS/OPD-58 | Landraces local collection | West Bengal India |
| 302 | IC598267 | DPS/OPD-60 | Landraces local collection | West Bengal India |
| 303 | IC598270 | DPS/OPD-66 | Landraces local collection | West Bengal India |
| 304 | IC598660 | TR - 64 | Landraces local collection | Champawat, Uttarakhand, India |
| 305 | IC615001 | KP-1859 | Landraces local collection | Pratapgarh, Rajasthan, India |
| 306 | IC615004 | KP-1863 | Landraces local collection | Pratapgarh, Rajasthan, India |
| 307 | IC73199 | NI-5643 | NA | Nashik, Maharashtra, India |
| 308 | IC75217 | KSML-3 | NA | Uttar Pradesh, India |
| 309 | IC75242 | HD-1962/E 4870/3/ K 65/5/SKA/6/UP 262 | NA | Uttar Pradesh, India |
| 310 | IC78766 | C-2427 | NA | Uttar Pradesh, India |
| 311 | IC82139 | DCB-492 | Landraces local collection | Sirohi, Rajasthan, India |
| 312 | IC82167 | DCB-541 | Landraces local collection | Dungarpur, Rajasthan, India |
| 313 | IC82432 | BDJ-I-123 | Landraces local collection | Hamirpur, Himachal Pradesh, India |

NA: Not available

**Table S2** Twenty-five salt tolerance QTNs identified in control condition using two or more than two multi-locus GWAS models.

| **Trait** | **QTN** | **Marker** | **Marker position (bp)** | **Allele** | **LOD score** | **'-log10(P)'** | **r2 (%)** | **Method** |
| --- | --- | --- | --- | --- | --- | --- | --- | --- |
| Chlorophyll content index (CCI) | Q.CCI-E1-1B | Affx-92649084 | 1B: 571704707 | G/A | 3.11-5.16 | 3.81- 5.96 | 2.27- 4.85 | 2, 4, 6 |
|  | Q.CCI-E2-6B | Affx-92570230 | 6B:696638770 | A/G | 4.06- 7.05 | 4.81- 7.92 | 7.70- 12.02 | 5, 4 |
| Green leaf area (GLA) | Q.GLA-E1-1A | Affx-92157471 | 1A:503993416 | C/T | 3.04- 3.86 | 3.74- 4.61 | 6.73- 7.92 | 4, 6 |
|  | Q.GLA-E1-1B | Affx-92122554 | 1B:492301285 | G/A | 3.99- 5.62 | 4.74- 6.44 | 4.48- 9.12 | 3, 4, 6 |
|  | Q.GLA-E1-2D | Affx-92399712 | 2D:585210854 | A/G | 3.02- 4.73 | 3.72-5.52 | 2.84- 9.02 | 1, 2, 5, 4, 6 |
|  | Q.GLA-E1-3A | Affx-92375804 | 3A:538639421 | C/T | 4.34-11.47 | 5.11-12.44 | 5.58- 16.33 | 1, 2, 6 |
|  | Q.GLA-E1-3B.1 | Affx-92953740 | 3B:723678106 | C/T | 3.29- 3.64 | 4.00- 4.38 | 1.33- 1.92 | 1, 2, |
|  | Q.GLA-E1.E2-4A | Affx-92170517 | 4A:737096657 | G/A | 4.30- 6.21 | 5.07- 7.05 | 4.59- 12.15 | 1, 3, 2, 3 4, 6 |
|  | Q.GLA-E1-5A.1 | Affx-92187251 | 5A:610217061 | G/A | 3.04- 3.30 | 3.74- 4.01 | 2.19- 2.79 | 1, 3 |
|  | Q.GLA-E1-5A.2 | Affx-92823494 | 5A:11786146 | C/T | 3.19- 4.43 | 3.90- 5.21 | 3.45- 5.40 | 1, 2, 5 |
|  | Q.GLA-E1-6B | Affx-92416453 | 6B:673635966 | T/G | 3.75- 4.23 | 4.49- 4.99 | 3.71- 4.90 | 2, 6 |
|  | Q.GLA-E1-6D.1 | Affx-92765994 | 6D:4410712 | A/G | 3.21- 4.54 | 3.92- 5.32 | 2.91-2.95 | 3 ,6, |
|  | Q.GLA-E1.E2-1D | Affx-92143230 | 1D:439868227 | C/T | 3.79- 5.60 | 4.53- 6.42 | 5.76- 10.65 | 2, 4 |
|  | Q.GLA-E2-3B.2 | Affx-92799999 | 3B:613071895 | C/T | 3.45- 6.42 | 4.18- 7.27 | 6.34- 11.63 | 5, 4 |
|  | Q.GLA-E2-6D.2 | Affx-92333705 | 6D:466953095 | G/C | 4.84- 4.74 | 5.52- 5.63 | 8.61- 23.89 | 1, 2 |
| Dry biomass (DB) | Q.DB-E1-1A | Affx-92159054 | 1A:520933199 | C/G | 3.88-6.52 | 4.62-7.37 | 5.35-5.69 | 5, 4 |
|  | Q.DB-E1-1B.1 | Affx-92766091 | 1B:14030253 | C/T | 3.96-17.15 | 4.71-18.20 | 6.04-23.69 | 1, 2, 3, 5, 4, 6 |
|  | Q.DB-E1-2D | Affx-92280827 | 2D:568940612 | A/G | 3.70-4.87 | 4.43-5.66 | 9.94E-07- 2.15 | 2, 5 |
|  | Q.DB-E1-3B | Affx-92209376 | 3B:278328740 | C/G | 3.15- 3.67 | 3.86- 4.40 | 8.27- 12.44 | 1, 4 |
|  | Q.DB-E1-6B.1 | Affx-92209457 | 6B:690968996 | G/C | 3.71- 6.05 | 4.45- 6.89 | 5.13-8.48 | 1, 2, 4, 6 |
|  | Q.DB-E1-7B | Affx-92919740 | 7B:403701530 | G/T | 3.38- 5.10 | 4.10- 5.90 | 2.75- 16.64 | 1, 3, 4 |
|  | Q.DB-E2-1B.2 | Affx-92563021 | 1B:10018025 | A/C | 3.34- 4.96 | 4.06- 5.76 | 1.79- 7.13 | 1, 3, 5, 4 |
|  | Q.DB-E2-1D | Affx-92748807 | 1D:445590609 | C/T | 3.08- 5.33 | 3.78- 6.14 | 1.52- 5.02 | 1, 5 |
|  | Q.DB-E2-6B.2 | Affx-92760068 | 6B:11540123 | T/C | 3.67- 5.19 | 4.41- 5.99 | 2.00E-04- 6.72 | 2, 3, 4 |
|  | Q.DB-E2-6A | Affx-92407505 | 6A:18007863 | T/C | 4.16- 6.71 | 4.92- 7.56 | 5.75- 11.65 | 1, 3 |

1: mrMLM; 2: FASTmrMLM; 3: FASTmrEMMA; 4: pKWmEB; 5: pLARmEB, 6: ISIS EM-BLASSO

**Table S3** Nineteen salt tolerance QTNs identified in treatment condition using two or more than two multi-locus GWAS models.

| **Trait** | **QTN** | **Marker** | **Marker position (bp)** | **Allele** | **LOD score** | **'-log10(P)'** | **r2 (%)** | **Method** |
| --- | --- | --- | --- | --- | --- | --- | --- | --- |
| Chlorophyll content index (CCI) | Q.CCI-E1-4D | Affx-92722153 | 4D:131268361 | G/C | 3.75-3.80 | 4.49- 4.54 | 2.00E-04- 5.81 | 2, 5 |
|  | Q.CCI-E1-5D.1 | Affx-92847476 | 5D:152734775 | A/G | 3.18- 3.18 | 3.88- 3.88 | 0.02- 0.04 | 2, 5 |
|  | Q.CCI-E1-6B | Affx-92238744 | 6B:574633571 | C/T | 3.08- 3.47 | 3.78- 4.19 | 5.98E-06-1.47E-07 | 5, 6 |
|  | Q.CCI-E2-5D.2 | Affx-92553843 | 5D:334697005 | C/A | 4.36- 4.49 | 5.12- 5.27 | 7.28- 11.51 | 4, 6 |
| Green leaf area (GLA) | Q.GLA-E1.E2-2B.1 | Affx-92775164 | 2B:9863211 | G/C | 3.68-4.12 | 4.41-4.87 | 3.68-6.11 | 1, 2, 3 |
|  | Q.GLA-E1.E2-5D | Affx-92918236 | 5D:537222235 | C/T | 3.02- 7.79 | 3.72- 8.67 | 6.05-10.64 | 1, 2, 3, 5, 6 |
|  | Q.GLA-E1-7B | Affx-92751769 | 7B:670443615 | C/T | 5.79- 6.59 | 7.40- 7.44 | 6.46- 8.86 | 5, 4 |
|  | Q. GLA-E2-2B.2 | Affx-92654322 | 2B:667805353 | G/A | 3.84- 5.74 | 4.58- 6.57 | 9.28- 21.20 | 2, 5, 4, 6 |
|  | Q. GLA-E2-4B | Affx-92322907 | 4B:660532402 | T/C | 3.12-4.35 | 3.82- 5.12 | 1.48- 1.60 | 2, 5, 4 |
|  | Q. GLA-E2-5A | Affx-92660442 | 5A:673405475 | T/G | 6.26- 10.03 | 7.10- 10.96 | 10.24- 14.14 | 2, 5, 4, 6 |
| Dry biomass (DB) | Q.DB-E1-1B | Affx-92750182 | 1B:573274254 | C/T | 3.35- 4.78 | 4.07- 5.57 | 7.64- 12.06 | 1, 4, 6 |
|  | Q.DB-E1-3A | Affx-92836429 | 3A:756305479 | G/A | 4.56-4.69 | 5.34- 5.47 | 14.20- 21.7 | 1, 4 |
|  | Q.DB-E1-4B | Affx-92478756 | 4B:40233959 | C/T | 3.68- 5.31 | 4.41- 6.12 | 0.29- 5.61 | 1, 2, 4 |
|  | Q.DB-E1-5A | Affx-92494400 | 5A:609868464 | C/T | 3.56-3.91 | 4.29- 4.66 | 0.00- 7.60 | 3, 4 |
|  | Q.DB-E1-5D | Affx-92119261 | 5D:469720080 | C/A | 3.47- 6.13 | 4.19- 6.97 | 6.92E-06- 0.29 | 2, 5 |
|  | Q.DB-E1-6B | Affx-92209457 | 6B:690968996 | G/C | 3.08- 3.54 | 3.78- 4.26 | 7.83E-07- 1.93 | 3, 5 |
|  | Q.DB-E2-1A | Affx-88639832 | 1A:480199288 | A/G | 3.09-3.52 | 3.79- 4.24 | 0.04- 5.09 | 2, 3 |
|  | Q.DB-E2-2B | Affx-92345045 | 2B:137610030 | C/T | 3.33- 3.46 | 4.04- 4.18 | 3.59- 8.21 | 1, 2 |
|  | Q.DB-E2-7B | Affx-92542799 | 7B:732254008 | C/A | 4.17- 4.92 | 4.93- 5.72 | 0.47- 7.37 | 5, 4 |

1: mrMLM; 2: FASTmrMLM; 3: FASTmrEMMA; 4: pKWmEB; 5: pLARmEB, 6: ISIS EM-BLASSO

**Table S4** Gene categories and number of genes detected within the LD

| **S.No.** | **Category** | **Number of genes** |
| --- | --- | --- |
| 1. | Transcription factors & regulators | 49 |
| 2. | Protein kinases & signaling components | 35 |
| 3. | Ubiquitin–proteasome system | 37 |
| 4. | Defense & resistance proteins | 21 |
| 5. | Metabolic enzymes (general metabolism, P450s, transferases, etc.) | 336 |
| 6. | Fe(II)/2-oxoglutarate dioxygenases (Fe2OG) | 3 |
| 7. | Transporters & membrane proteins | 21 |
| 8. | RNA processing & chromatin-related proteins | 25 |
| 9. | Others / Uncharacterized | 3 |
|  | **Total** | **530** |
